# Abiotic and Herbivory Combined Stress in Tomato: Additive, Synergic and Antagonistic Effects and Within-Plant Phenotypic Plasticity

**DOI:** 10.3390/life12111804

**Published:** 2022-11-07

**Authors:** Rosa Vescio, Roberta Caridi, Francesca Laudani, Vincenzo Palmeri, Lucia Zappalà, Maurizio Badiani, Agostino Sorgonà

**Affiliations:** 1 Department Agraria, University Mediterranea of Reggio Calabria, Località Feo di Vito, 89122 Reggio Calabria, Italy; 2 Department of Agriculture, Food and Environment, University of Catania, Via Santa Sofia 100, 95123 Catania, Italy

**Keywords:** within-plant phenotypic plasticity, combined stresses, additive, antagonistic and synergic effects, VOCs

## Abstract

Background: Drought, N deficiency and herbivory are considered the most important stressors caused by climate change in the agro- and eco-systems and varied in space and time shaping highly dynamic and heterogeneous stressful environments. This study aims to evaluate the tomato morpho-physiological and metabolic responses to combined abiotic and herbivory at different within-plant spatial levels and temporal scales. Methods: Leaf-level morphological, gas exchange traits and volatile organic compounds (VOCs) profiles were measured in tomato plants exposed to N deficiency and drought, *Tuta absoluta* larvae and their combination. Additive, synergistic or antagonistic effects of the single stress when combined were also evaluated. Morpho-physiological traits and VOCs profile were also measured on leaves located at three different positions along the shoot axes. Results: The combination of the abiotic and biotic stress has been more harmful than single stress with antagonistic and synergistic but non-additive effects for the morpho-physiological and VOCs tomato responses, respectively. Combined stress also determined a high within-plant phenotypic plasticity of the morpho-physiological responses. Conclusions: These results suggested that the combined stress in tomato determined a “new stress state” and a higher within-plant phenotypic plasticity which could permit an efficient use of the growth and defense resources in the heterogeneous and multiple stressful environmental conditions.

## 1. Introduction

Owing to sessile nature, plants are continually exposed to abiotic (mainly drought, heat and salinity) and biotic (pathogens and herbivory) stresses whose intensity and frequency are expected to be increased by climate change. The effects of these stresses and how the plants respond to these stressful factors, taken individually, have been extensively studied at both the morpho-physiological and molecular scale [1,2] and plant community level [3,4]. However, under field condition, these various biotic and abiotic factors are constantly changing during the plant life cycle and, above all, co-occur in nature [5]. Hence, the plants have to make decisions about fine-tuning their responses to allocate resources efficiently for responding to the more serious and different threats at any given point in time. Different studies have uncovered that plants evoke a “unique response” to the combination of abiotic and biotic stresses compared to the single stress [5,6,7,8] revealing that the plants’ responses to combined stress pointed out “a new stress state” with mostly non-additive effects (i.e., synergistic or antagonistic). For example, insect herbivory antagonized the heat responses in tomato [9], whereas, in this same plant species, drought stress synergized the emission of specific VOCs in combination with aphid herbivory [10] as well with the larvae of green alder sawfly (*Monsoma pulveratum*) in *Alnus glutinosa* [11]. Drought and simulate herbivory combination synergized some morphological traits of *Pinus sylvestris*, while other ones were antagonized [12]. In addition to the “new stress state”, the plants’ responses to the stress are strictly dependent on the plant traits, genotypes, species, and type, intensity, frequency and duration of the stress suggesting that more investigation is needed for a better understanding of the abiotic and pest herbivore interaction, the least studied among stress combinations.

The plants’ responses to the individual abiotic and biotic stress have been observed to show a modulation (induced and constitutive) with a strong spatio-temporal component (local or systemic, transient or permanent) that determined a high “within-plant variation”. For example, the spatial scale of herbivore-induced changes can range from localized at the site of attack [13] to systemic throughout the entire plant or tissue type [14,15]. Additionally, the light and heat gradients determined different responses within the tree canopy [16] or the nutrient deficiency caused different morpho-physiological responses among the different root types [17,18]. The temporal scale of the plant responses can also vary: rapid or long term, ontogeny-modulated [19] and, in some cases, even trans-generational responses are evoked for herbivory [20] as well as for abiotic stress [21]. The multiple ecological role of the ‘within-plant’ variation was recently pointed out in the adaptation to individual abiotic and biotic gradients [22] as well as in the alteration of plant–antagonist interactions [23] so much so that it was proposed as “functional trait itself” whose influences on ecosystem functioning are still neglected [24]. In spite of this important role, the within-plant variation in response to the combined stress has not been investigated so far, to the best of our knowledge.

Since 2006, the tomato production of the Mediterranean region has been under attack by a newly introduced insect, *Tuta absoluta*, whose larvae feed on leaves, stems and fruits causing severe damage to the tomato with decreases in production both in the field and greenhouse [25]. Previous studies revealed that the low nitrogen levels and drought stress inputs to tomato negatively affected the biological traits of *T. absoluta* [26,27] but also demonstrated that the N deficiency and drought could be unfavorable to the tomato plants, suggesting that the trade-off between negative impact on *Tuta* pest and plant growth should be evaluated.

In this framework, experiments were set up to study the spatial and temporal expressions of the morpho-physiological and metabolic responses of the tomato plants to the single and/or combined abiotic (drought+N deficiency) and biotic stress (herbivory by *T. absoluta*). In particular, the present study investigates the following questions: (1) Are the morpho-physiological responses to individual stresses different from the combined ones in tomato plants? (2) Are additive, synergistic or antagonistic effects in the combined stress? (3) Do the tomato responses to the single and combined stress occur at between- or within-plant levels?

## 2. Materials and Methods

### 2.1. Experimental Procedure and Plant Material

The present study was carried out on hydroponically-grown tomato plants (*Solanum lycopersicum* L., cultivar nano S. Marzano) and consisted of two separate experiments, addressing different but interrelated questions.

The first experiment, denoted as the ‘synergic, antagonistic and additive effects’, aimed to determine the tomato responses to the abiotic and biotic stress, single or in combination, and their temporal evolution, and also to evaluate whether the responses to the combined stress were the results of additive, synergistic or antagonistic effects of the single stress. For this purpose, we evaluated the effects of the drought plus N deficiency (abiotic stress, ABIO), or herbivory by *T. absoluta* (biotic stress, BIO) and their combination (combined stress, COMB) with the time of exposure (0, 1, 3 and 8 days) on the morphological (leaf fresh and dry weight), physiological [water content, photosynthesis, stomatal conductance, transpiration rate and intrinsic water use efficiency (iWUE)] and metabolic traits (VOCs profile). The fresh and dry weight of the leaf are traits directly related to the plant status, while the leaf water content was strictly correlated with the plant drought tolerance [28] but also with the plant palatability [29]. The gas exchange traits (photosynthesis, stomatal conductance, transpiration and iWUE) are involved in the plant responses to drought and N deficiency [30,31] and further the photosynthesis is “…a plant-driven response to the perception of stress rather than a secondary physiological response to tissue damage…” highlighting strict interactions between photosynthesis, reactive oxygen species (ROS) and hormonal signaling pathways for the plant responses to insect herbivory [32]. Finally, the VOCs, as direct and indirect defense, are emitted by plants subject to both abiotic and biotic stress [33].

Tomato plants (*Solanum lycopersicum* L., cultivar nano S. Marzano) (provided by BAVICCHI S.p.a., Perugia, Italy) were exposed to the abiotic (nitrogen limitation and drought stress simulated by the use of polyethylene glycol (PEG)) and biotic stress (two first instar larvae placed in a leaf), or their combination and were considered as ‘stress condition’. The control plants (CTR) were maintained at optimal N concentration, no drought and herbivory, considered as the ‘optimal condition’. For the morpho-physiological analysis, we used a randomized block design in which the entire experiment yielded a total of 4 (treatments) × 4 (time of exposure) × 2 (block) × 2 (replications) = 64 samples. The block was introduced because we used two experiments at two different times. A completely randomized design was used for the VOCs profiling, in which the entire experiment was constituted by 4 (treatments) × 4 (time of exposure) × 3 (replicates) × 3 (measurements) = 144 samples. The replicates for the VOCs were obtained in three different experiments.

The second experiment, denoted as the “within-plant phenotypic plasticity”, aimed to evaluate the within-plant variation of the tomato morpho-physiological and metabolic traits and how this within-plant phenotypic plasticity changed with the treatment conditions (optimal, abiotic, biotic and combined stress). For this aim, the foliar morpho-physiological and metabolic traits were evaluated on mature leaves located at three different positions along the shoot axes for each treatment. For each treatment, we used a completely randomized design in which the entire experiments yielded a total of 3 (leaves) × 1 (time of exposure) × 3 (replicates) = 9 samples. For the gas exchanges traits only, we took two measurements for each leaf, hence the experiments provided 3 (leaves) × 1 (time of exposure) × 2 (measurements) × 4 replicates = 24 samples.

The Figure S1 reported the experimental protocol schedule of both experiments including the plant growth, treatments and analysis.

### 2.2. Tomato Growth Conditions

Tomato seeds were surface sterilized for 15 min in 10% (*v*/*v*) sodium hypochloride, rinsed with tap water and then germinated in a Petri dish (diameter 90 mm) on filter paper with 0.1 mM CaSO_4_. After 7 d of germination [7 days after germination (DAG)], six seedlings of uniform size were transferred into each of eight hydroponic units, each containing 4.5 L of the following aerated nutrient solution (http://www.haifa-group.com/files/Guides/tomato/Tomato.pdf (accessed on 8 February 2016) at 50% strength and adjusted to pH 6.0 with 0.1 M potassium hydroxide: 5 mM KNO_3_, 1 mM NH_4_NO_3_, 1.44 mM MgSO_4_, 3.99 mM Ca(NO_3_)_2_, 0.97 mM KH_2_PO_4_, 1 mM K_2_SO_4_, 25 μM H_3_BO_3_, 50 μM KCl, 2 μM MnSO_4_, 4 μM ZnSO_4_·7H_2_O, 0.5 μM CuSO_4_·5H_2_O, 0.5 μM (NH_4_)Mo_7_O_24_·4H_2_O, 20 μM EDTA iron(III) sodium salt.

The hydroponic units were placed in a growth chamber at 24 °C, 14 h photoperiod; photon flux rate of 300 µmol m^−2^ s^−1^; 70% relative humidity (RH).

After 7 days (14 DAG), the nutrient solution was brought to 100% strength and the plants of each pot were thinned to four for the morpho-physiological analysis while they were left to six for VOCs analysis. The nutrient solution was renewed every 2 days.

### 2.3. Insect Rearing

The tomato leafminer *Tuta absoluta* (Lepidoptera: Gelechiidae) colony was maintained in climatic chambers (25°C, RH 70%, 16 h light). It was kept in cages (Bugdorm^®^—60 × 60 × 60 cm) containing tomato plants. Sugar and water were provided *ad libitum* to adults in rearing cages.

### 2.4. Abiotic Stress and Herbivory Treatment

At 28 DAG, six hydroponic units continued to receive the same nutrient solution as previously described in growth conditions, while in two hydroponic units 5% (*w*/*v*) polyethylene glycol 8000 (Sigma PEG8000, Sigma Aldrich, St. Louis, MO, USA) and 1 mM nitrogen were added for simulating the drought stress and nitrogen deficiency, respectively (ABIO group). The final PEG concentration was gradually achieved by the addition of 2.5% (*w*/*v*) PEG8000 every two days. The osmotic potential of the solutions, measured by an osmometer (Freezing point osmometer, Osmomat 3000, Gonotec, Berlin, Germany), was −0.55 MPa for 5% PEG and −0.05 MPa for the control solution (0% PEG). To obtain 1 mM N for the nitrogen deficiency, the NH_4_NO_3_ was not added and the KNO_3_ and Ca(NO_3_)_2_ were reduced to 1 mM and 0.5 mM, respectively. In order to balance K and Ca, the K_2_SO_4_ and CaSO_4_ were increased to 3 mM and the 3.5 mM, respectively. Preliminary experiments were run to ascertain that the selected PEG8000 and N concentrations did not prejudice plants’ survival.

At 42 DAG, the hydroponic units were treated as follows for obtaining the whole set of treatments (Figure S1):

(1) two hydroponic units were renewed with the optimal nutrient solution (CTR group);
(2) two hydroponic units were renewed with the nutrient solution with N deficiency and PEG (ABIO group);
(3) two hydroponic units received the optimal nutrient solution but the plants were infested with *Tuta* larvae to induce the biotic stress (BIO group);
(4) two hydroponic units maintained the same nutrient solution with N deficiency and PEG and, in addition, the plants were infested by *Tuta* larvae (COMB group).

The plant infestation was obtained by placing two first instar larvae of *Tuta* in the 1st fully-developed leaf (with five leaflets) from the bottom and, to avoid larvae escaping, each infested leaf was then bagged with a nylon mesh of 4.7 cm diameter (Figure S2). We added herbivorous insects to plants after 14 days of abiotic stress treatment in order to simulate the effects of a pest outbreak which are predicted to become more frequent with climate change [34].

### 2.5. First Experimental: Synergic, Antagonistic and Additive Effects

#### 2.5.1. Tomato Samplings and Measurements

At 0 (42 DAG), 1 (43 DAG), 3 (45 DAG) and 8 days from the treatments (50 DAG), the measurements/samplings were realized in order to simulate the early, intermediate and late responses, respectively. Gas exchange measurements were carried out on the terminal leaflet of the first fully-developed leaf (in presence of larvae, we used lateral leaflets) while the whole plants were used for the morphological analysis. Consecutively, three leaves for each treatment and time of exposure were sampled for the VOCs.

#### 2.5.2. Gas Exchange Measurements

A calibrated portable photosynthesis system (LI-6400; LI-COR, Inc.; Lincoln, NE, USA) was used to measure the net CO_2_ assimilation rate (A) (μmol (CO_2_) m^−2^ s^−1^), stomatal conductance (gs) (mol H_2_O m^−2^ s^−1^), and the transpiration rate (T) (mmol H_2_O m^−2^ s^−1^). These gas exchange parameters were measured at 500 cm^3^ min^−1^ flow rate, 26 °C leaf temperature, CO_2_ concentration 400 μmol (mol air)^−1^ (controlled by CO_2_ cylinder), and 1200 µmol m^−2^ s^−1^ of photosynthetically active radiation supplied by the LED light source in the leaf chamber. Each measurement was made with a minimum and maximum wait time of 120 and 200 s, respectively, and matching the infrared gas analyzers for 50 μmol (CO_2_) mol (air)^−1^ difference in the CO_2_ concentration between the sample and the reference before every change of plants. The leaf-to-air vapor pressure difference (VPD) was set to 1.5 kPa, and continuously monitored around the leaf during measurements. It was maintained at a constant level by manipulating the humidity of incoming air as needed. All measurements were performed in the growth chamber.

Finally, the intrinsic water use efficiency (iWUE) was calculated as the rate of photosynthesis (A) divided by the rate of stomatal conductance to water (gs) [35].

#### 2.5.3. Morphological Measurements

All the leaves of the plants were harvested, immediately weighed to obtain the leaf fresh weight (LFW, g) and then placed in an oven at 70 °C for 2 days to determine the leaf dry weight (LDW, g).

Finally, the leaf water content (LWC, %) was calculated as the following, as reported in Jin et al. [36]:

Leaf Water content (%) = (LFW − LDW)/LFW × 100(1)

#### 2.5.4. VOCs Analysis

VOCs from three leaves per treatment and time of exposure were profiled by headspace–solid phase microextraction (HS/SPME) method. One leaf was sealed in a 20 mL hermetic vial with butyl lid and allowed to incubate for 20 min at room temperature. The fiber (50/30 μm DVB/CAR/PDMS) (Supelco^®^, Bellefonte, PA, USA), previously conditioned according to the supplier’s instructions, was inserted into the headspace of the vial containing the sample and allowed to adsorb leaf volatiles for 20 min. The volatiles were then desorbed by placing the fiber for 6 min into the injection port of the gas chromatography–mass spectrometry (GC-MS) system. All the SPME sampling and desorption conditions were identical for all the samples. Blanks were performed before first SPME extraction and randomly repeated during each series of measurements.

GC-MS analysis of VOCs were performed with a Thermo Fisher TRACE 1300 (Trace 1300, Thermo Fisher Scientific, Waltham, MA, USA) gas chromatograph equipped with a DB-5 capillary column (30 m × 0.25 mm; coating thickness = 0.25 μm, with 10 m of pre-column) coupled to a Thermo Fisher ISQ LT ion trap mass detector (ISQ LT, Thermo Fisher Scientific, Waltham, MA, USA) (emission current: 10 microamps; count threshold: 1 count; multiplier offset: 0 volts; scan time: 1.00 second; prescan ionization time: 100 microseconds; scan mass range: 30–300 *m/z*; ionization mode: EI).

GC–MS data were obtained under the following analytical conditions: carrier gas Helium (He 99.99%); flow rate 1 mL/min; spiltless. The initial oven temperature was 60 °C for 3 min, after which it was raised to 240 °C at 6 °C/min, and finally isothermal for 3 min. The injection port, transfer line, and ion source were kept at 250 °C, 250 °C, and 260 °C, respectively.

Qualitative identification of VOCs was performed using GC–MS reference libraries (NIST x.0). Linear retention indices (LRI) were determined from the retention times of a series of n-alkane mixture (C8-C20, Sigma Aldrich, Milan, Italy) analyzed under the same conditions reported above [37]. Percentages of the studied compounds were calculated from the peak areas in the total ion chromatograms. The relative abundance of each volatile with respect to the total amount of released compounds was estimated from its peak area against the total ions chromatogram, and expressed as a percentage, after subtracting possible contaminants.

#### 2.5.5. Statistical Analysis

##### Morpho-Physiological Data

By SPSS Inc. V. 10.0, 2002 (SPSS Inc., Evanston, IL, USA), all the morpho-physiological parameters were analyzed by two-way ANOVA with the treatment (Tr) (CTR, ABIO, BIO and COMB), time of exposure (Ti) and block (Bl) as main factors and the TrxTi as interaction. Then, Tukey’s test was used to compare the means of all the parameters of each Tr and Ti. All data were tested for normality (Kolmogorov–Smirnoff test) and homogeneity of variance (Levene median test) and, where required, the data were transformed.

##### VOCs Data

The VOCs dataset was elaborated by using the R statistical software 3.5 [38].

Differences among treatments, time of exposure and TrxTi interaction were inferred through PERMANOVA multivariate analysis (999 permutations) using the package *vegan*. Pairwise comparisons were calculated using a custom script and correcting *p* values using the False Discovery Rate (FDR) method.

In order to identify VOCs key predictors that could constitute a molecular signature identifier among the treatments within each time of exposure, we used a preliminary unsupervised (Principal Component Analysis, PCA) and then supervised analysis (Sparse Projection to Latent Structure–Discriminant Analysis, sPLS-DA) by using the *mixOmics* package [39]. Statistical algorithms are detailed in Rohart et al. [39] and they account for multiple comparisons inherent in biomarker datasets, where multiple classification features are considered for a relatively small number of specimens (*p* >> n). In particular, the sPLS-DA procedure constructs artificial latent components of the predicted dataset (VOCs Table denoted X (N × P)) and the response variable (denoted Y with categorical information of samples, e.g., CTR, ABIO, BIO and COMB). To predict the number of latent components (associated loading vectors) and the number of discriminants, for sPLS-DA, we used the *perf.plsda()* and *tune.splsda()* functions, respectively. We finetuned the model using five-fold cross-validation repeated 10 times to estimate the classification error rates employing two metrics, overall error rates and balanced error rates (BER), between the predicted latent variables with the centroid of the class labels (categories considered in this study) and specifying the *max.dist* (which gave the minimal classification rate in this study).

##### Calculation of Additive, Synergistic or Antagonistic Effects in Combined Stress

To determine whether stress combination yielded additive, synergistic or antagonistic effects respect to each of the single stress alone, we used the method of Bansal et al. [12]. To such aim, we compared the observed effects (Ob) with expected additive effects (Ex) for the plants exposed to the abiotic stress and herbivory combination (COMB) at 3 and 8 days of treatments, only. The Ob effect sizes were calculated as the absolute value of:

Ob = (ob − xCTR)/xCTR (2)

where Ob is the value of each measured trait in each plants and treatment and xCTR is the mean value of the same trait measured in CTR plants.

For each of the traits considered, the Ex additive effect sizes for the COMB treatment were defined in two steps by first determining and then summing the independent effects (In) of each treatment. The In effect sizes were calculated as the absolute value of:

Ind = (xstress − xCTR)/xCTR (3)

where xstress is the mean values of a given trait in the presence of a single stress, and xCTR is the corresponding mean value in CTR plants. Then, the Ex additive effect size for the COMB treatment was calculated by using a multiplicative risk model [40], that is the sum of the two In effects minus their product. Finally, the Ex additive values for COMB plants were compared to the actual Ob additive effects. In particular, we calculated a mean difference (±95% confidence interval) between the effect sizes of Ob and Ex was for COMB plants. When Ob-Ex > 0 and the lower 95% confidence limit was greater than zero, then the impact from the combination of both stressors was classified as synergistic. Antagonistic effects were defined when the Ob-Ex < 0 and the upper 95% confidence limit was less than zero. Finally, we classified additive effects when the 95% confidence interval crossed the zero line.

### 2.6. Second Experiment: Within-Plant Phenotypic Plasticity

#### 2.6.1. Tomato Samplings and Measurements

The tomato samplings and measurements were carried out at 8 days from the treatments (50 DAG) in the leaves located at three different positions along the shoot axes: basal (B), intermediate (I) and apical leaf (A) belonging to the first, second and third node, respectively. Preliminary experiments using phloem dying as reported in Orians et al. [41] resulted that the apical leaf, but not the intermediate one, is linked to the basal one via vasculature connections (Figure S3). On such basis, we also considered the basal, intermediate and apical leaves as the local (L), no-orthostichous (nO) and orthostichous leaf (O), respectively. The basal/local leaf was used for placing the first instar larvae of *T. absoluta* for the experimental infestation.

Measurements for the gas exchanges traits were carried out on two opposite leaflets of the basal/local (B/L), intermediate/noOrthostic (I/noO) and apical/orthostic leaf (A/O) and the same leaves were subsequently collected for the morphological analysis.

All the morpho-physiological analyses were carried out as in the first experiment.

#### 2.6.2. Statistics

##### Within-Plant Variance of the Morpho-Physiological Traits

The within-plant variance of the morpho-physiological traits was evaluated as in Zywiec et al. [42].

In order to estimate the partitioning of total variation of the morpho-physiological traits among- and within-treatments, we conducted a linear mixed models with treatments and plant nested within-treatments as random effects using the whole-plant data. The variance partitions among- and within-treatments and tests on the statistical significance of variance components were conducted using restricted maximum likelihood (REML).

In order to verify the effects of each treatments on morpho-physiological traits of different leaves within the plants, we analyzed the within-plant variation by applying a hierarchical partition to divide total variance into two levels of variation: among plants and among the leaves in the same plants (leaf nested within plant). All levels were considered as random effects, as required for variance partitioning. Analyses were conducted with the mixed procedure of SPSS. The replicate obtained for each leaflets sample allowed us to estimate measurement error and thus assess the variance component and statistical significance (Wald Z and *p* values) of this component between- and within-individual plants.

##### Morpho-Physiological Data

By SPSS Inc. V. 10.0, 2002 (SPSS Inc., Evanston, IL, USA), all the morpho-physiological parameters were analyzed by one way ANOVA with Tukey’s test as post-hoc test (*p* < 0.05).

## 3. Results and Discussion

### 3.1. Are the Morpho-Physiological Responses to Individual Stresses Different from the Combined Ones? Are Additive, Synergistic or Antagonistic Effects in the Combined Stress?

**Figure 1:**
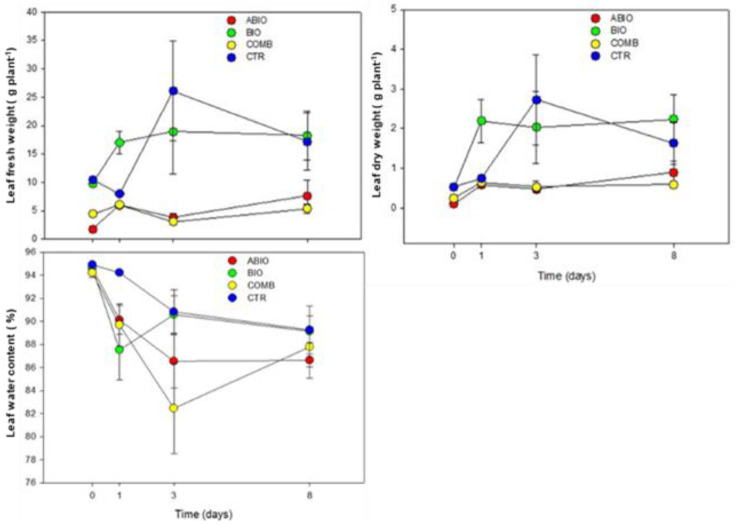
Morphological traits. Leaf fresh (g), dry weight (g) and leaf water content (%) of tomato plants treated with different stress: drought stress plus N deficiency (ABIO), infestation by the insect *Tuta absoluta* (BIO) and their combination (COMB) for different times of exposure (0, 1, 3 and 8 days). Unstressed control plants (CTR). Each value and its error bar indicate the mean and the standard error of the mean, respectively (N = 4).

**Figure 2:**
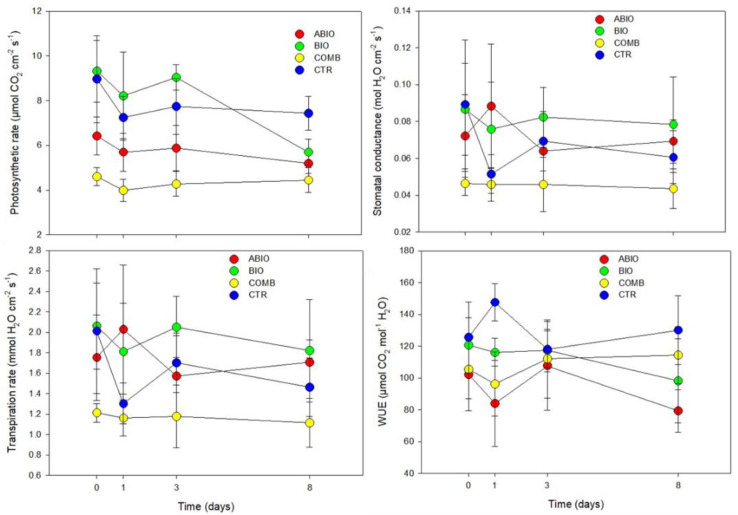
Gas exchange parameters. Photosynthetic rate (µmol CO_2_ m^−2^ s^−1^), stomatal conductance (mol H_2_O m^−2^ s^−1^), transpiration rate (mmol H_2_O m^−2^ s^−1^) and intrinsic water use efficiency (µmol CO_2_ mol^−1^ H_2_O) of the leaves of tomato plants treated with different stress for different time of exposure (0, 1, 3 and 8 days). Acronyms as in Figure 1. Each value and its error bar indicate the mean and the standard error of the mean, respectively (N = 4).

The morpho-physiological results clearly indicated an opposite pattern in the response of the tomato plants to the single stresses with the ABIO treatment showing a more negative impact than the BIO one with respect to the CTR plants (Figure 1 and Figure 2).

In particular, the LFW, LDW, LWC, A, and iWUE were significantly reduced in the ABIO plants with respect to the control, whereas no significant differences were observed in the presence of herbivory except than for leaf water content and iWUE (Figure 1 and Figure 2; Table 1 and Table 2).

It is known that the drought stress, either alone [43] or in combination with N deficiency [44], reduced the A, g_s_ and LWC with negative consequence for the leaf growth of tomato plants which arise from established molecular mechanisms [45]. As observed, the BIO treatment did not produce modification of the morpho-physiological traits in comparison to the control (Figure 1 and Figure 2; Table 1 and Table 2) and this no response to the herbivory falls in the highly variable effects observed in different plant–insect combinations. For example, the leaf dry to fresh mass ratio was not changed by *Monsoma pulveratum* feeding on *Alnus glutinosa* [11] but a weak negative effect in the soybean-natural herbivory interaction was observed [46]. Further, the net photosynthesis was found to be either sharply reduced [47], or even increased [48] or even not modified [49]. In the present study, the infestation of *Tuta absoluta* could probably have caused ‘indirect effects’ in leaf tomato such as increase of the photosynthesis and water losses by transpiration rate, resulting in reduced iWUE and leaf water content as also observed in soybean-Japanese beetles and corn earworm caterpillars interactions [49]. However, in a previous study concerning the *Tuta*–tomato interactions, a reduction in leaflet growth was pointed out [50].

Although the plant responses to the individual abiotic stress and herbivory infestation are well understood, the information concerning the effects of stress combination is, in most of the cases, scanty or even absent. In the present study, the combination among the N deficiency and drought, on one side, and herbivory by *Tuta*, on the other side, caused the highest reduction of the tomato morpho-physiological traits respect to the control plants (Figure 1 and Figure 2; Table 1 and Table 2). This overstate effect of the combined stress could be due to the interactive responses determined by cross-talk in hormonal signaling and by the transcriptional modulation of defense-related genes. Indeed, in the interaction between *Solanum dulcamara* and the herbivory by specialist *Leptinotarsa decemlineata*, the antagonism of the specific herbivory-induced salycilic acid on the jasmonic acid (JA) was found to prevail over the synergism of the specific drought-induced abscissic acid (ABA) with consequent reduction of the defense responses observed at transcriptional levels (increase in the cell wall components and secondary metabolism) [51]. Moreover, in tomato plants subjected to both drought and herbivory by *Spodoptera exigua*, an adaptive response was observed by a transcriptional activation of the genes related to the photosynthetic machinery and chlorophyll biosynthesis causing, as a consequence, a reduction of the secondary metabolite production [51].

The temporal evolution of the plant responses to environmental stresses is fundamental for the success of the plant adaptation, although such aspect has been comparatively less studied. In the present work, differently to the single stress, the COMB treatment reduced the LFW and LDW at 3 days from the stress treatments, while the LWC, A, g_s_ and iWUE respond faster, being already evident at 1 day of treatment (Figure 1 and Figure 2; Table 1 and Table 2). It is likely that the combined stress in tomato plants rapidly activated the stomatal closure, to reduce the water losses, causing in turn a reduction of the photosynthetic process accompanied by a decrease in the synthesis of defense-related metabolites and all this subsequently translated into a lower leaf growth. However, this morpho-physiological pattern, although faster, could have been the final result of the signaling and molecular network which is instead activated in very rapid responses (within seconds and minutes) as observed in different abiotic- and biotic-stressed plants [21].

Figure 1 and Figure 2 and Table 1 and Table 2 also show that abiotic stress and herbivory by *Tuta* more negatively affected the physiological traits than the morphological ones. The plant physiological plasticity is more related to an enhanced ability to exploit the transient environmental resources, such as water and nutrient patches, or to produce the defense responses (secondary metabolites) to the herbivory attack at low cost by short-term adjustments [52,53,54]. Conversely, the plant morphological plasticity is more resource-intensive and hence more functional to long-term plant adaptation [52,54].

**Figure 3:**
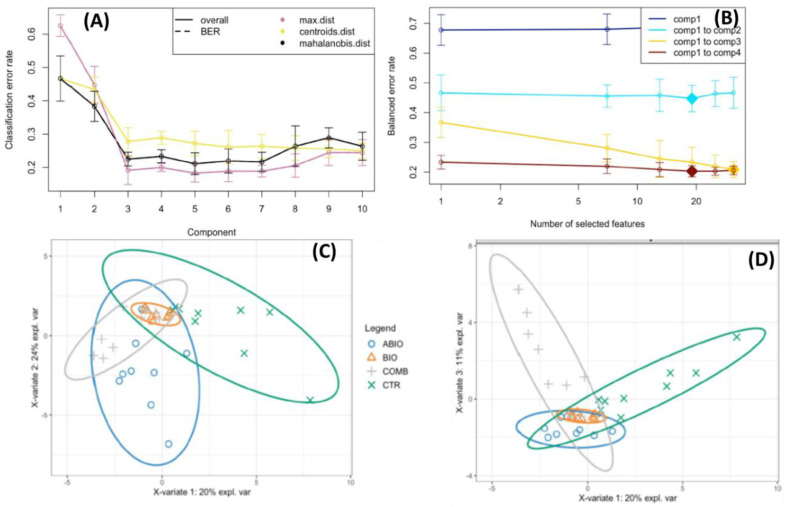
Sparse Projection to Latent Structure–Discriminant Analysis (sPLS-DA) of volatile organic compounds emitted from the leaves of tomato plants exposed to different stress (ABIO, BIO, COMB, see acronym sin Figure 1), or not stressed (CTR) for 3 days of exposure. (**A**) Choosing the number of components in sPLS-DA by performance test. Mean classification by overall and balanced error rate (5 cross-validation averaged 50 times) for each sPLS-DA component. (**B**) Choosing the number of volatiles for each sPLS-DA components by tuning test. Estimated classification balanced error rates for volatile dataset (5 cross-validation averaged 50 times) with respect to the number of selected volatiles for the sparse exploratory approaches. (**C**,**D**) sPLS-DA sample plot for the different components using 95% confidence ellipses. (**C**) Component 1 vs. Component 2, (**D**) Component 1 vs. Component 3.

The VOCs emission is an important plant defense process in response to the herbivory attack [55], as well as abiotic stress [56]. HS/SPME GC-MS analysis revealed forty-five volatile compounds emitted by the tomato leaves exposed to the single (abiotic and biotic) and combined stress (Table S1). In particular, the volatile profile was mostly characterized by mono- (24% of the total) and sesquiterpenes (44%), although hydrocarbons (11%), ester (9%), alcohol (5%), ether (5%) and aldehyde (2%) were also present (Table S1). Volatile terpenoid metabolites have been recognized as having a range of specific roles in plant/environment and plant/plant interactions [57] and, in particular, in the direct and indirect defense of tomato plants against the herbivory [58]. Table S2 reported the volatiles from each treatment and time of exposure on the basis of % area of each peak over the total area of the chromatogram. In order to test the influence of the treatments and time of exposure on the volatile profiling, we run a multivariate approach that included, first, the Permanova, and then the PCA and sPLSDA that allowed to visualize the differences among the groups and to select informative and relevant volatiles. Permanova analysis indicated that the treatments, the time of exposure and their interaction determined significant differences in the volatilome of tomato plants (Table S3). Pairwise comparison among the treatments within each time of exposure revealed that the volatile profile of COMB plants was different from control ones at 3 and 8 days of exposure, while in the BIO plants such differences emerged only at 8 days of exposure (p_adjusted_ < 0.05; Table S4). Since differences among the treatments became evident after the two aforementioned times of exposure, we only used the volatiles dataset from the four treatments at 3 and 8 days of exposure to run the PCA. Figure S4 showed the results of PCA where it is apparent that this multivariate test was not able to separate the treatment groups owing to the high variability among samples. Therefore, data were further analyzed using sPLS-DA at each time of exposure (3 and 8 days). At 3 days of exposure, the performance step of the sPLSA-DA for the selection of the number of components suggested that three components were sufficient to sharply reduce the balanced error rate around 0.23 (Figure 3A). Further, the final model obtained by tuning process pointed out that Component 1, 2 and 3 comprised 25, 19 and 31 volatiles, respectively (Figure 3B), but with a scarce discrimination among the treatments, as highlighted by the sample plots on the first three components (Figure 3C,D).

**Figure 4:**
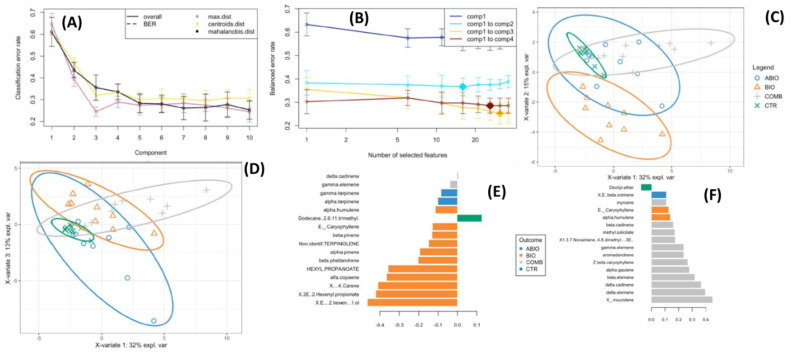
Sparse Projection to Latent Structure–Discriminant Analysis (sPLS-DA) of volatile organic compounds emitted from the leaves of tomato plants exposed to different stress (ABIO, BIO, COMB, see acronym sin Figure 1), or not stressed (CTR) for 3 days of exposure. (**A**) Choosing the number of components in sPLS-DA by performance test. Mean classification by overall and balanced error rate (5 cross-validation averaged 50 times) for each sPLS-DA component. (**B**) Choosing the number of volatiles for each sPLS-DA components by tuning test. Estimated classification balanced error rates for volatile dataset (5 cross-validation averaged 50 times) with respect to the number of selected volatiles for the sparse exploratory approaches. (**C**,**D**) sPLS-DA sample plot for the different components using 95% confidence ellipses. (**C**) Component 1 vs. Component 2, (**D**) Component 1 vs. Component 3. Contribution plots by loading weights of the volatiles selected for the Component 2 (**E**) and Component 1 (**F**) of the sPLS-DA. The color indicated the treatments for which the selected volatile has a maximal mean loading weight value.

At 8 days of exposure, three components were selected with a balanced error rate around 0.28 and with a molecular signature composed of 16, 16 and 31 VOCs selected on the first three components, respectively (Figure 4A,B). The sample plots on the three components put into evidence a discrimination among the treatments with 60% of total explained variability split up by 32%, 15% and 13% for the first, second and third components, respectively (Figure 4C,D).

In particular, plotting the first two components showed that the BIO treatment was sharply separated from the control and COMB ones by the second component (Figure 4C). Conversely, the first and third components scarcely discriminated among the treatments (Figure 4C,D). All 16 VOCs selected on the second component had a negative weight in the linear combination, and a subset of them, namely (E)-2 hexen-1 ol, (2E)-2-hexenyl propionate, (+)-4-carene, α-copaene, hexyl propionate, β-phellandrene, α-pinene, terpinolene, β-pinene, E β-caryophyllene, were found to be emitted at comparatively higher rates in BIO plants (Figure 4E). However, a higher emission was observed for dodecane, 2,6,11-trimethyl in CTR plants, the α-humulene and α-terpinene in ABIO ones, and γ-elemene and δ-cadinene in COMB ones (Figure 4E). The alcohol (E)-2 hexen-1-ol [59], and the two esters (2E)-2-hexenyl propionate [60] and hexyl propionate [61] are green leaf volatiles which are released in response to different stress conditions to aid in plant defense against herbivory and bacterial and fungal pathogens [62]. The (+)-4-carene, α-copaene, β-phellandrene, α-pinene, terpinolene, β-pinene and E β-caryophyllene are terpenes, a large family of organic compounds mostly involved in the plant defense. For example, the (+)-4-carene, α-copaene and β-phellandrene are the most abundant VOCs emitted by *Solanum* spp. in the presence of *Bactericera cockerelli* herbivory [63], and terpinolene and E β-caryophyllene were mainly produced by tomato leaves infested with *Trialeurodes vaporario-rum* [64]. Further, the α-humulene was found to be responsible of tomato repellence against *Bemisia tabaci* [65]. The dodecane, 2,6,11-trimethyl, which, on a comparative basis, was found to be highly emitted by the CTR plants of the present study (Figure 4E), could be regarded as a VOCs marker of healthy plant status, since this same evidence was obtained by Giunti et al. in olive plants [66]. Interestingly, the α-terpinene and δ-terpinene were found to be mainly discriminant of ABIO treatment (Figure 4E), thus confirming the results obtained in tomato plants (cv. Gan Liang Mao Fen 802 F1) fertilized with low levels of N [65] and in drought-stressed *Thymus vulgaris* plants [67]. Finally, the present study revealed the VOCs emitted by multi-stressed tomato plants. In particular, the γ-muurolene, δ-elemene, δ-cadinene, β-elemene, α-gauiene, z-β-caryophyllene, aromadendrene, γ-elemene, 1,3,7 Nonatriene 4,8 dimethyl (3E)-, methyl salicylate, β-cadinene and myrcene were the compounds constituting the VOCs blend emitted by tomato plants when exposed to combined N and drought stress with infestation by *Tuta absoluta* (Figure 4E,F). It should be noted that the γ-elemene and δ-cadinene were present in both sPLD-DA components while the other volatiles were only observed in the Component 1 that is the lesser discriminant (Figure 4E,F). In *Gossypium arboretum*, the δ-cadinene is a precursor of the cyclic secondary sesquiterpene aldehydes, including gossypol, that are insecticides [68]. Unlike δ-cadinene, the emission of γ-elemene was not modified in the tomato leaves exposed to pest attack [64]. Besides the δ-cadinene and γ-elemene, the other volatiles belonging to the component 1 were also of interest in plant responses to the abiotic and biotic stress. Methyl salicylate was observed to increase in double drought-stressed and aphid-infested tomato plants [10] but also in the drought-herbivory combination together with 1,3,7-nonatriene, 4,8-dimethyl-, (3E)-, with which methyl salicylate forms a couple of stress-specific VOCs [11]. The other volatiles released by the COMB plants of the present study were also present in the volatilome of different plant species exposed to the combination of two or more stresses [11,69,70].

### 3.2. Are Additive, Synergistic or Antagonistic Effects in the Combined Stress?

**Figure 5:**
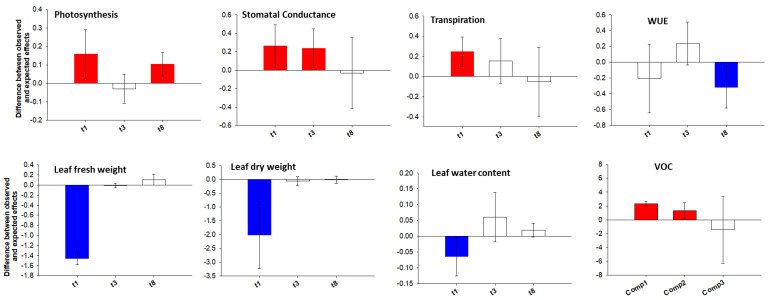
The combined impacts from abiotic (drought and N deficiency) and biotic stress (Infestation of *Tuta absoluta*) on morpho-physiological traits of tomato plants at 1, 3 and 8 days of treatment and VOCs emission at 8 days of treatment. The combined impact of single stressors was estimated as synergist (red color), additive (white color) or antagonistic (blue color) (greater than, equal to or less than expected effects, respectively, based on single stressor effect sizes). The vertical and error bars represent, respectively, the mean and the 95% confidence interval of the overall effect size difference between the observed and expected additive effects from combined abiotic and biotic stress on morpho-physiological and metabolic traits of tomato plants. The zero line represents the expected additive effects from combined stressors. When the means (and their 95% confidence limits) were higher than or less than the zero line, they were considered synergistic or antagonistic, respectively.

In analyzing the tomato morpho-physiological and metabolic responses to the combined stress, it is of interest to understand whether stress combination caused additive (i.e., equal to the sum of the single-stress effects), synergistic (i.e., higher than expected) or antagonistic effects (i.e., lower than expected) with respect to those caused by each single stress taken alone. This, in turn, would allow hypotheses to rise about the signaling pathways and molecular mechanisms underlying the plant strategy in the presence of simultaneous stress. In this respect, the additive, synergistic and antagonistic effects of abiotic (drought and N deficiency) and biotic stress in tomato plants were evaluated by the Bansal et al. method [12]. The reported results show that, in general, the physiological (photosynthesis, stomatal conductance and transpiration) and the metabolic (VOCs) traits pointed out more synergistic effects than morphological ones especially at an early time of exposure, i.e., at 1 and 3 days (Figure 5).

In particular, a synergic effect (Figure 5) was observed for the reduction of the physiological traits (photosynthesis, stomatal conductance and transpiration) (Figure 2) and for the increase in the VOCs emission (Table S2). The closure of the stomata is the first plant response to the water scarcity and it is mediated by ABA that orchestrates a network of stress-responsive metabolites and gene expression [71]. The ABA signaling pathways also interact with that of the JA one, the phytohormone that activates the signaling cascades for regulating downstream transcriptional responses to the herbivory [72,73] and, furthermore, it was observed that MeJA signaling is overlapped with ABA signaling in guard cells [74]. In the present work, such interaction between the ABA and JA signaling pathways could have caused the synergistic negative effect on the physiological traits observed in COMB plants (Figure 5). However, besides the hormonal interactions, unique and novel molecular mechanisms were also found during the stress combination in several studies. For example, the transcriptome analysis revealed that a unique set of transcripts was altered in response to the combination of drought and nematode infection [75], drought, heat stress and virus [76], infection by *Botrytis cinerea*, herbivory by chewing larvae and drought stress [77]. In the present study, the observed synergic effect of stress combination on the decrease of both photosynthetic and transpiration rates could have been determined by the stomatal closure in addition to the herbivory-induced resource reallocation to chemical defense [78] that determined more intense dark respiration [79].

A synergic effect (Figure 5) was also involved in the increase of the metabolic traits such as the VOCs emission in COMB-treated tomato plants (Table S2). This synergic effect could be due to diverse reasons. First, the improvement of the formation reactive oxygen species (ROS) by drought stress [80] and nutrient deficiency [81] that could sensitize the VOCs response. Indeed, it is known that the VOCs are emitted by early signaling events involving the ROS during the herbivory [82]. Secondly, the abiotic stress and herbivory by *Tuta* could have increased the biosynthesis of VOCs both via hormone cross-talking and higher resource reallocation to chemical defense. For example, the ABA and JA, the main phytohormones respectively involved in the plant response to the drought and herbivory, interact among each other via molecular cross-talk [72,73,74] and the reallocation of plant resources to defense by modification of the gene expression profiles after herbivory was also observed [83]. Third, the increased VOCs biosynthesis in presence of both stresses could have caused their accumulation inside the leaf, leading to the formation of a steep partial pressure gradient between the atmosphere and substomatal cavities along which the VOCs could be highly emitted.

Unlike the physiologic and metabolic traits, the results obtained for the morphological traits suggested an early antagonistic effect (at 1 day of treatment) in COMB plants, evolving thereafter towards additivity (Figure 5). This result could be explained by the fact that COMB plants prioritized their responses towards herbivory. Indeed, the COMB plants aimed to reduce their leaf water content more in the presence of *Tuta* rather than under ABIO ones compared to the CTR through a sharp increase of the leaf dry weight (Figure 1). Why? The water and the dry weight are strictly and negatively linked to the plant palatability towards the pest [84]; hence, in the presence of combined stress, tomato plants might have redirected the allocation of their resources towards the formation of carbon-based secondary compounds such as lignin and fiber contents which contribute to leaf toughness and reduce palatability [85].

### 3.3. Do the Tomato Responses to the Single and Combined Stress Occur at Between- or Within-Plant Levels?

**Figure 6:**
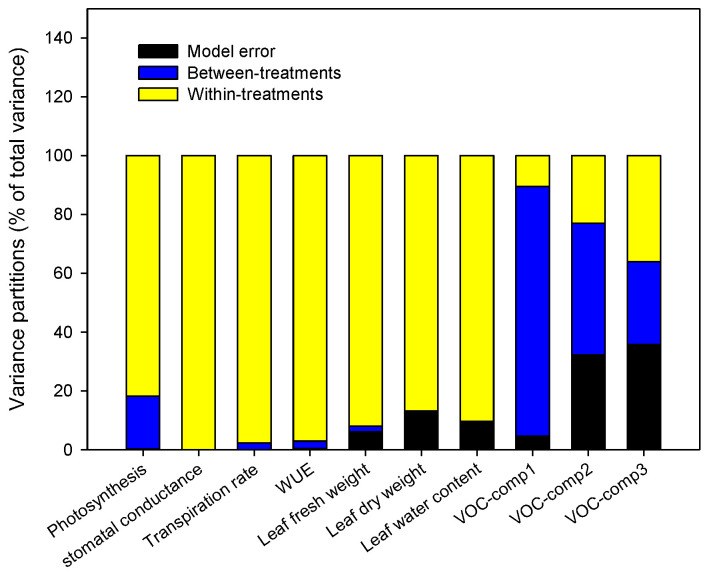
Dissection of total variance components of the morpho-physiological traits and VOCs responses at 8 days of treatments (control, abiotic stress, biotic stress, combined stress). Considering all treatments pooled, the contributions of treatment (blue color) and within-treatment (yellow color) level to the total variance in mean of the morpho-physiological traits and VOCs responses in the four treatments considered were estimated by linear mixed models.

Recent studies pointed out the importance of the within-individual variation of the plant responses to the abiotic and biotic stress rather than between-individual for the ecology at individual, population, and community levels [86,87]. For example, it was pointed out that a higher within-plant variation of the morpho-physiological responses permitted an improvement of the exploitation of the heterogeneously distributed resources such as light, CO_2_, nutrient [24,88], an optimization of the cost-expensive defenses against herbivory and pathogens [89], and an alteration of plant–antagonist interactions [23]. In this respect, first, we assessed whether the among-treatments variance of the morpho-physiological traits and VOCs profiles of the tomato plants is higher than within-treatment ones, and then, we evaluated the between- and within-plant variance for each treatment. To do this, we used the morpho-physiological traits and VOCs observed at 8 days, that is the time at which wider and higher modifications of these traits became evident (Table 1 and Table 2; Figure 1, Figure 2 and Figure 4). The contribution of the among- and within-treatment levels to the total variance in mean of the morpho-physiological traits and VOCs responses to the treatments considered was estimated by linear mixed models and statistically tested by restricted maximum likelihood (REML) (Figure 6).

**Figure 7:**
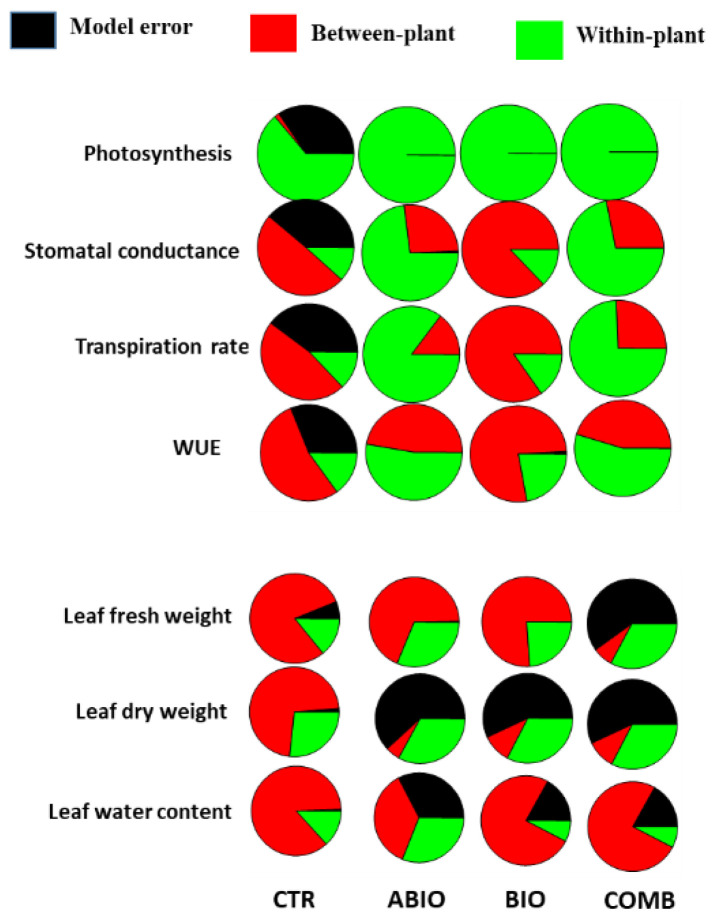
Nested within-treatment variance partitions (% of the total) in the morpho-physiological responses of tomato plants to different abiotic and biotic stress. The between-plant variance comprises plant within treatment (red color) and the within-plant variance involved the leaves within plant within treatment (green color). The black color indicated the model error.

Figure 6 indicated that most variance occurred at the within-treatments level, especially for the morpho-physiological traits but not for the VOCs. Hence, unlike the morpho-physiological traits, the emission of the VOCs was more dependent on the stress treatments rather than the individual plants. For such reason, only the morpho-physiological traits were used in the analysis of within-plant variation that was conducted applying a hierarchical partition to divide total variance into two levels of variation: among plants and among the leaves within the same plant. Such analysis was performed for each single treatment in order to verify its effects on within-plant phenotypic plasticity. The variance partitions varied substantially among the different treatments and traits considered (Figure 7).

In general, the physiological traits pointed out a higher within-plant variance (average 54%) while the morphological ones showed more between-plant variance (average 50%) (Figure 7). Why did the individual tomato plants modify more their leaf physiological traits than morphological ones within their shoot? This was likely owing to the physiological traits being comparatively less expensive, in terms of metabolic resources, and their modification could have been comparatively faster in response to the abiotic and biotic stress; for example, rapid local and systemic responses through specific signaling pathways have been observed in the presence of light stress [90], or herbivory [91], or during acquisition of heat tolerance [16]. Among the treatments, the stresses induced a higher within-plant variance than growing under optimal conditions: ABIO (average 58%), COMB (average 53%), BIO (average 31%) and CTR (average 22%) (Figure 7). The within-plant variation under stressful conditions could allow a better ability to exploit the stress-induced transient changes in environmental and soil resources [53,92] and the optimization of the defenses against herbivory [93].

**Figure 8:**
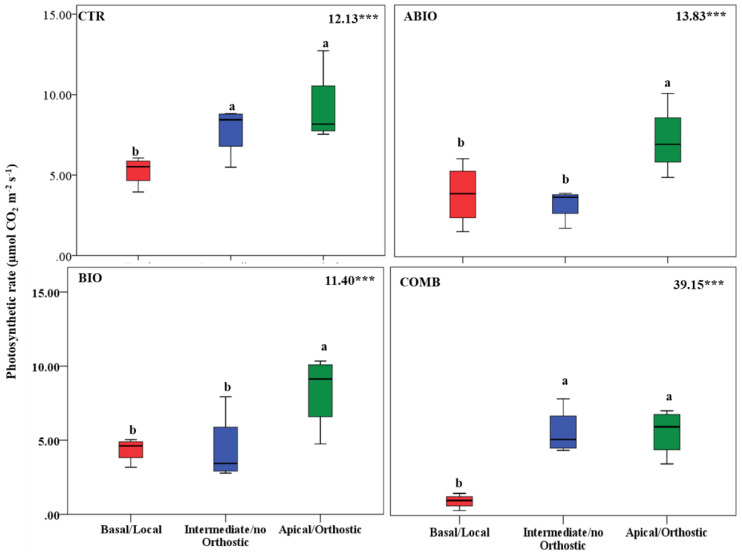
Photosynthetic rate of three different leaves of tomato plants exposed for 8 days at diverse stresses (abiotic (ABIO); biotic (BIO); combined (COMB)). No stresses (CTR). The box plot indicated the minimum, first quartile, median, third quartile, and maximum value. Different letters indicated significant difference among the mean groups (N = 8; *p* < 0.05 test of Tukey). The values within each panel indicated the F statistic with the *p* values (*** *p* < 0.001) derived from one-way ANOVA.

**Figure 9:**
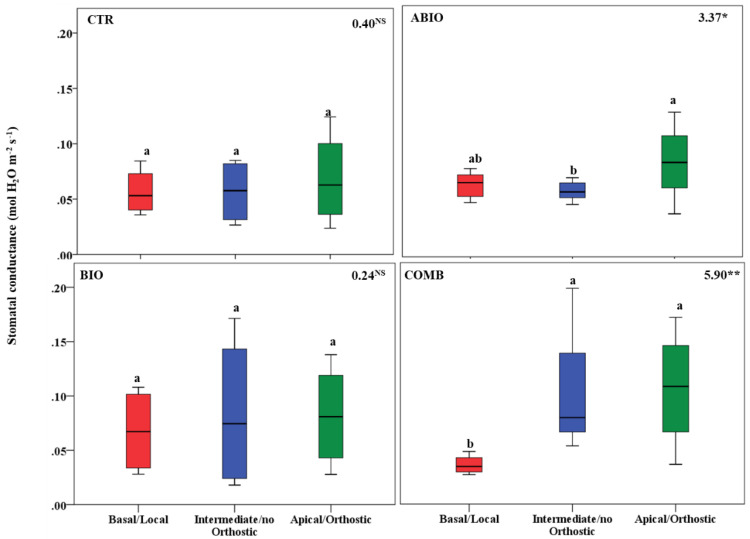
Stomatal conductance of three different leaves of tomato plants exposed for 8 days at diverse stresses (abiotic (ABIO); biotic (BIO); combined (COMB)). No stresses (CTR). The box plot indicated the minimum, first quartile, median, third quartile, and maximum value. Different letters indicated significant difference among the mean groups (N = 8; *p* < 0.05 test of Tukey). The values within each panel indicated the F statistic with the *p* values (* 0.05 < *p* < 0.01; ** 0.01 < *p* < 0.001; NS not significant) derived from one-way ANOVA.

**Figure 10:**
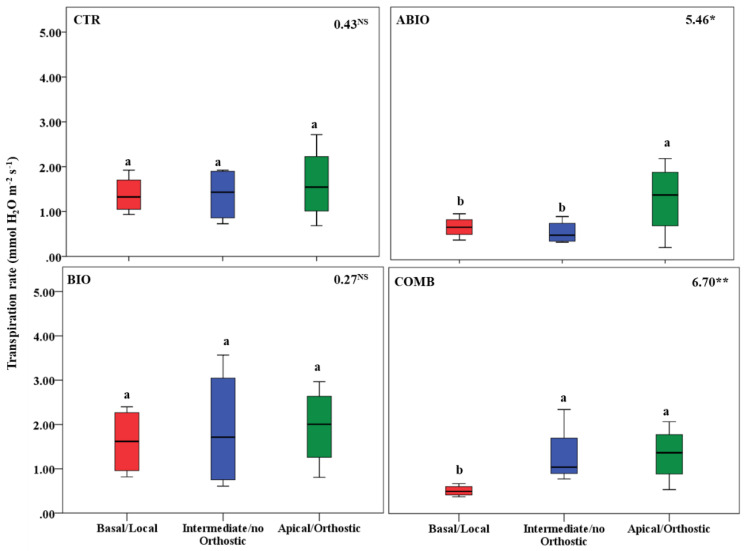
Transpiration rate of three different leaves of tomato plants exposed for 8 days at diverse stresses (abiotic (ABIO); biotic (BIO); combined (COMB)). No stresses (CTR). The box plot indicated the minimum, first quartile, median, third quartile, and maximum value. Different letters indicated significant difference among the mean groups (N = 8; *p* < 0.05 test of Tukey). The values within each panel indicated the F statistic with the *p* values (* 0.05 < *p* < 0.01; ** 0.01 < *p* < 0.001; NS not significant) derived from one-way ANOVA.

**Figure 11:**
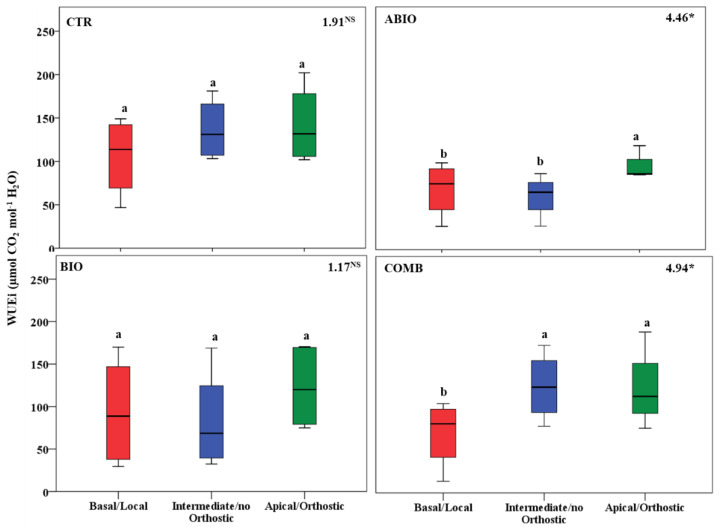
iWUE of three different leaves of tomato plants exposed for 8 days at diverse stresses (abiotic (ABIO); biotic (BIO); combined (COMB)). No stresses (CTR). The box plot indicated the minimum, first quartile, median, third quartile, and maximum value. Different letters indicated significant difference among the mean groups (N = 8; *p* < 0.05 test of Tukey). The values within each panel indicated the F statistic with the *p* values (* 0.05 < *p* < 0.01; NS not significant) derived from one-way ANOVA.

**Figure 12:**
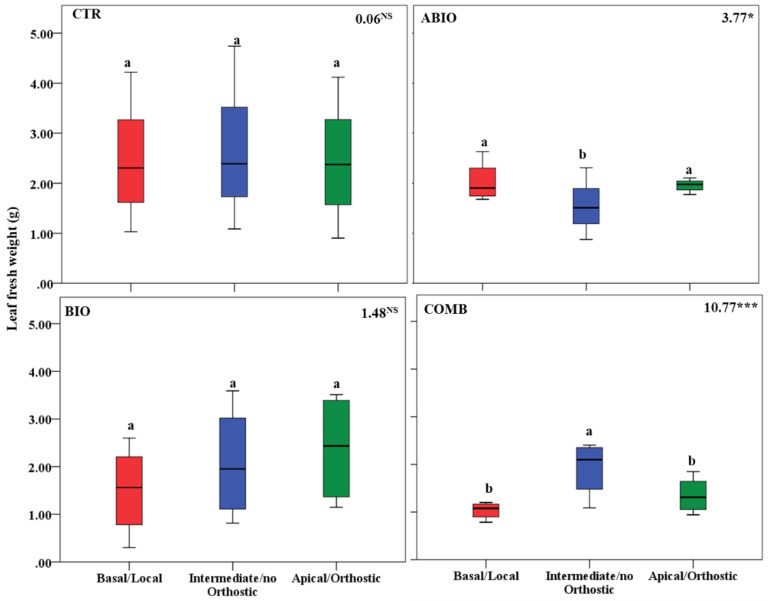
Leaf fresh weight of three different leaves of tomato plants exposed for 8 days at diverse stresses (abiotic (ABIO); biotic (BIO); combined (COMB)). No stresses (CTR). The box plot indicated the minimum, first quartile, median, third quartile, and maximum value. Different letters indicated significant difference among the mean groups (N = 8; *p* < 0.05 test of Tukey). The values within each panel indicated the F statistic with the *p* values (* 0.05 < *p* < 0.01; *** *p* < 0.001; NS not significant) derived from one-way ANOVA.

**Figure 13:**
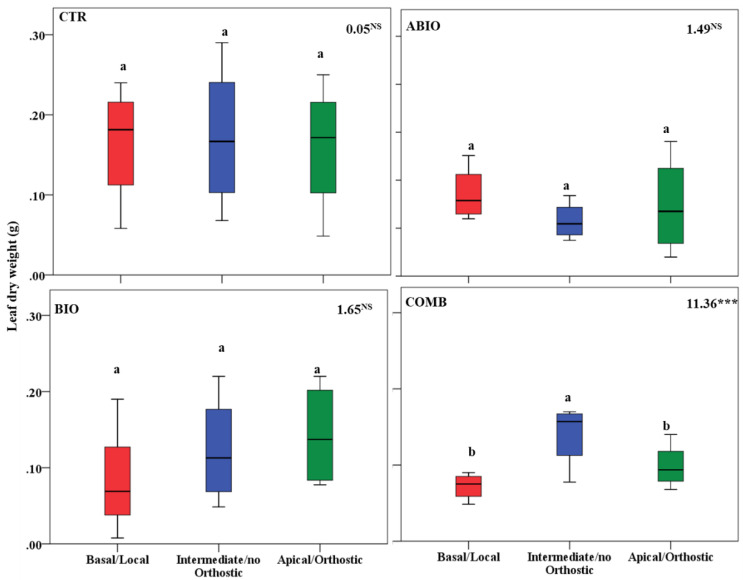
Leaf dry weight of three different leaves of tomato plants exposed for 8 days at diverse stresses (abiotic (ABIO); biotic (BIO); combined (COMB)). No stresses (CTR). The box plot indicated the minimum, first quartile, median, third quartile, and maximum value. Different letters indicated significant difference among the mean groups (N = 8; *p* < 0.05 test of Tukey). The values within each panel indicated the F statistic with the *p* values (*** *p* < 0.001; NS not significant) derived from one-way ANOVA.

**Figure 14:**
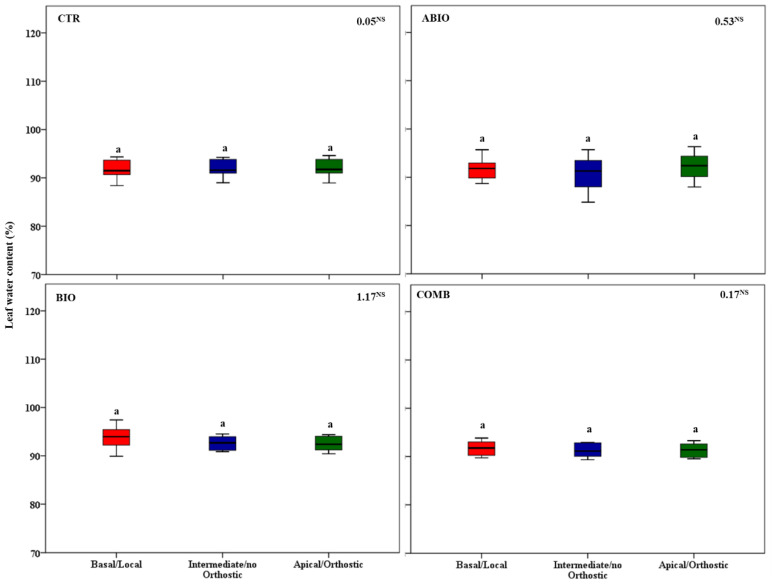
Leaf water content of three different leaves of tomato plants exposed for 8 days at diverse stresses (abiotic (ABIO); biotic (BIO); combined (COMB)). No stresses (CTR). The box plot indicated the minimum, first quartile, median, third quartile, and maximum value. Different letters indicated significant difference among the mean groups (N = 8; *p* < 0.05 test of Tukey). The values within each panel indicated the F statistic with the *p* values (NS not significant) derived from one-way ANOVA.

By considering that each treatment pointed out an important within-plant variation for the morpho-physiological traits, we asked if a well-defined spatial pattern of these responses among the leaves of the tomato shoot could be revealed. In this respect, by one-way ANOVA, we compared the morpho-physiological responses to each treatment on three mature leaves located at three different positions (basal (B), intermediate (I) and apical leaf (A) placed at first, second and third node, respectively) along the shoot axes on the morpho-physiological traits for each treatments. Further, the B, I and A leaf can be also considered as local (L), no-orthostichous (nO) and orthostichous leaf (O), respectively, because the apical leaf, but not the intermediate, is linked to the basal one by vasculature connection (Figure S3). Hence, such leaf selection allowed to evaluate which among the vascular (L vs. O vs. nO) or architectural patterns (B vs. I vs. A) caused the within-plant phenotypic variability. Figure S5 depicted the vascular or architectural pattern or no pattern of the tomato responses to each treatments. The Figure 8, Figure 9, Figure 10, Figure 11, Figure 12, Figure 13 and Figure 14 showed the results of gas exchanges (A, gs, T, and iWUE) and morphological traits (LFW, LDW, LWC) for each treatment.

An overall result is that, unlike the CTR and BIO, the ABIO and the COMB treatments significantly modified all the morpho-physiological traits among the three tomato leaves, except the LWC and A (Figure 8, Figure 9, Figure 10, Figure 11, Figure 12, Figure 13 and Figure 14). Further, by comparing the spatial patterns of plant responses (Figure S5), the physiological responses (A, g_s_, T, and iWUE) to the ABIO and COMB treatments suggested an architectural pattern while the morphological ones pointed out a vascular pattern (Figure 8, Figure 9, Figure 10, Figure 11, Figure 12, Figure 13 and Figure 14). The abiotic stressors are known to strongly influence the plant photosynthetic traits in relation to the leaf position/age, reflecting an architecture pattern, in order to preserve the highly valuable tissues, such as the young leaf [94]. The vascular pattern of the LFW and LDW in response to the COMB treatment could have been due to the ABA-JA cross-talking signaling pathways [72,73,74] which could have occurred between the two vascularly connected leaves (local and orthostic). In this respect, we could hypothesize that the *Tuta* larvae placed on the local leaf of tomato plants could have triggered, by vascular connection, these hormonal signaling pathways which could have redirected the photosynthetic resources towards the defense compounds rather than leaf growth ones. This kind of hormone cross-talk signaling interaction among the different vascularly connected leaves within the plant has already been observed. For example, abiotic stresses antagonized the immune responses by ABA-SA hormonal interaction in older leaves of Arabidopsis, but such effect was suppressed in the younger leaf through a signaling component of the SA pathway [95].

## 4. Conclusions and Future Directions

The present study has been addressed to answer questions related to the tomato responses in the presence of combined abiotic stress (drought and N deficiency) and herbivore infestation, based on the prediction that crop plants will have to face an increased incidence of both detrimental factors in a changing climate. A first result of the present study was that, respect to each single stress taken alone, the combination of drought, N deficiency and *Tuta* infestation caused a stronger negative impact on the tomato morpho-physiological traits and induced a specific VOCs blend. It is likely that hormone cross-talking regulating the signaling and metabolic systems of the plant responses could be assumed as an explanation. Interestingly, and unlike each single stress, the VOCs blend induced by stress combination contained, among others, the homoterpene 4,8-dimethyl-1,3,7-nonatriene, known to be a rare and fundamental plant alarm volatile, as well as methyl salicylate, a well-known herbivore-induced plant volatile which attracts natural enemies and affects herbivore behavior.

A second result and main outcome of the present study was the relatively rapid responses of the tomato plants to the COMB treatment and the synergistic effects for the physiological and VOCs responses, but antagonistic for the morphological ones. In this respect, no-additive effects of the single stress in tomato response to the combined stress were put into evidence. This remarkably suggests that a “new stress state” characterized by specific signaling pathways and gene expression, and probably orchestrated by hormonal interactions, could be evoked in tomato plants stressed by the combination of drought, N deficiency and *Tuta* infestation.

Finally, except for the VOCs emission, the stressful conditions induced a higher within-plant variance in tomato with the abiotic and combined stress being the most influential. The increase of the stress-induced variability of the morpho-physiological responses within the tomato plants is of interest, because it supports the view that a high within-plant variance could be of help for the defense against herbivore infestation and for maximizing the exploitation efficiency of scarce soil resources.

In deployment of the stress-adaptation strategies by the modification of the morpho-physiological traits, the plants use a signaling network consisting of several interacting pathways such as, for example, the production and detoxification of reactive oxygen species (ROS) and calcium-, phytohormone-, and MAPK-signaling pathway regulated at the multi-genic level. These metabolic pathways in plants subjected to the combination of different abiotic and biotic stress resulted in “shared” or “unique” responses with respect to those pointed out in presence of the single stress. Future research direction should be based on the investigation of these complex molecular networks that produce a “new stress state” tailored for stress combinations. By omic technologies, it is possible to identify specific genes involved in such shared and unique responses under combined stresses, which is an important step toward developing potential stress tolerance traits useful for providing multiple stress resistance to the crops.

## Figures and Tables

**Table 1:** Results of two-way ANOVA [Treatment (Tr), time (Ti), block (Bl), TrxTi interaction (TrxTi)] on the morphological traits. Abbreviations as in Figure 1. Statistics: F- and *p*-values. Within each morphological traits and time of exposure, the different letters indicated statistical differences among the means of the treatments (*p* < 0.05, test of Tukey).

Parameters	Statistics		Time (Ti)			
		Treatments (Tr)	t0	t1	t3	t8
Leaf fresh weight	Tr 10.92 ***	ABIO	a	a	b	b
	Ti 1.40 ^NS^	BIO	a	a	a	a
	Bl 6.08 *	COMB	a	a	b	b
	TrxTi 1.06 ^NS^	CTR	a	a	a	a
Leaf dry weight	Tr 8.87 ***	ABIO	a	a	b	b
	Ti 2.91 *	BIO	a	a	a	a
	BI 17.17 ***	COMB	a	a	b	b
	TrxTi 1.26 ^NS^	CTR	a	a	a	ab
Leaf water content	Tr 3.16 *	ABIO	a	ab	ab	a
	Ti 12.01 ***	BIO	a	b	a	a
	BI 52.15 ***	COMB	a	b	b	a
	TrxTi 1.50 ^NS^	CTR	a	a	a	a

* 0.05 < *p* < 0.01; *** *p* < 0.001; NS not significant.

**Table 2:** Results of two-way ANOVA [Treatment (Tr), time (Ti), block (Bl) TrxTi interaction (TrxTi)] on the gas exchanges traits. Abbreviations as in Figure 1. Statistics: F- and *p*-values. Within each gas exchanges traits and time of exposure, the different letters indicated statistical differences among the mean of the treatments (*p* < 0.05, test of Tukey).

Parameters	Statistics		Time (Ti)			
		Treatments (Tr)	t0	t1	t3	t8
Photosynthetic rate	Tr 17.60 ***	ABIO	a	b	bc	ab
	Ti 2.73 ^NS^	BIO	a	a	a	ab
	BI 20.74 ***	COMB	a	b	c	b
	TrxTi 0.76 ^NS^	CTR	a	ab	ab	a
Stomatal conductance	Tr 5.38 **	ABIO	a	a	ab	a
	Ti 0.60 ^NS^	BIO	a	a	a	a
	BI 61.82 ***	COMB	a	b	b	a
	TrxTi 0.57 ^NS^	CTR	a	ab	ab	a
Transpiration rate	Tr 6.94 **	ABIO	a	a	ab	ab
	Ti 0.79 ^NS^	BIO	a	a	a	a
	BI 55.73 ***	COMB	a	a	b	b
	TrxTi 1.13 ^NS^	CTR	a	a	ab	ab
iWUE	Tr 6.23 **	ABIO	a	b	a	b
	Ti 0.47 ^NS^	BIO	a	b	a	b
	BI 136.54 ***	COMB	a	b	a	ab
	TrxTi 1.52 ^NS^	CTR	a	a	a	a

** 0.01 < *p* < 0.001; *** *p* < 0.001; NS not significant.

## Data Availability

The authors declare that the data supporting the findings of this study are available within the article and Supplemental Materials, as well as from the corresponding author upon reasonable request.

## Supplementary Materials

The following supporting information can be downloaded at: https://www.mdpi.com/article/10.3390/life12111804/s1, Figure S1: Protocol schedule including tomato growth and treatments and plant sampling events, Figure S2: Trap for *Tuta absoluta*’s larva, Figure S3: Evaluation of the degree of vascular connectivity among leaves located in different position along the shoot. Figure S4: Principal component analysis applied to volatiles emission. Figure S5: Tomato responses to the experimental conditions resembling the vascular pattern or architectural pattern or no pattern (blue line). Table S1: Volatile organic compounds identified using gas chromatography/mass spectrometry (GC/MS), Table S2: Volatile organic compounds (Area %) from the leaves of tomato plants, Table S3: Permanova results of the VOCs emission, Table S4: PERMANOVA pairwise comparison between treatments.
