# Supplementary material for: Abiotic and Herbivory Combined Stress in Tomato: Additive, Synergic and Antagonistic Effects and Within-Plant Phenotypic Plasticity
Source: Life (Basel). 2022 Nov 7;12(11):1804. doi: 10.3390/life12111804 (PMC9699328; doi:10.3390/life12111804)
Supplement: Supplementary file 1 [file life-12-01804-s001.zip › Figure S1.pdf]

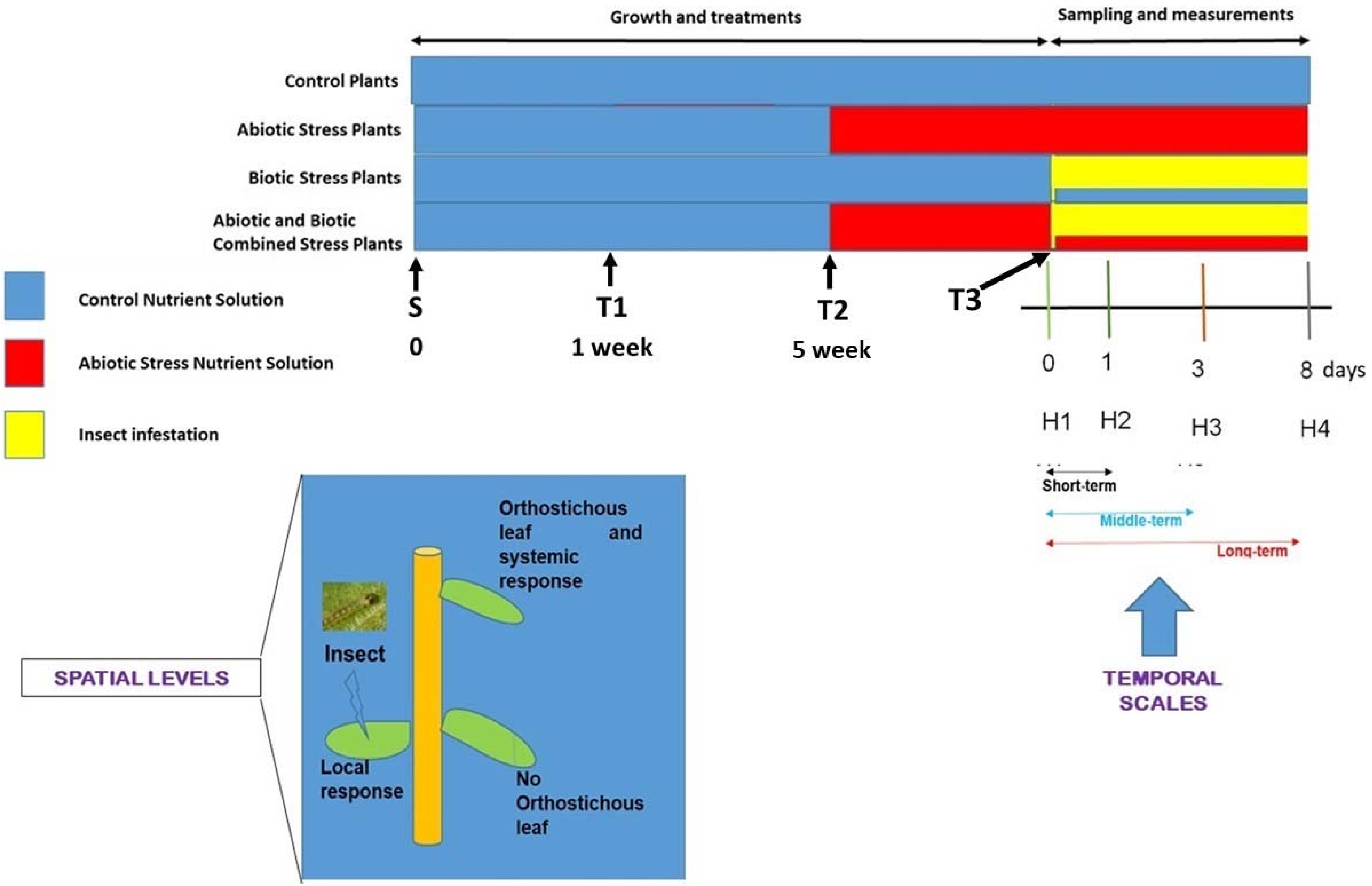

Figure S1 – Protocol schedule including tomato growth and treatments (S: plant seeding; T1: Plant transfer to hydroponic system; T2: abiotic treatment start; T3: *Tuta absoluta* larvae infestation) and plant sampling events (H1-H4: samplings and analysis). Analysis: morphological analysis (leaf fresh and dry weight, leaf water content), physiological (photosynthetic rate, stomatal conductance, transpiration rate and WUEi) and VOC profiling. The tomato responses were evaluated at temporal and spatial scales.
