# Supplementary material for: Abiotic and Herbivory Combined Stress in Tomato: Additive, Synergic and Antagonistic Effects and Within-Plant Phenotypic Plasticity
Source: Life (Basel). 2022 Nov 7;12(11):1804. doi: 10.3390/life12111804 (PMC9699328; doi:10.3390/life12111804)
Supplement: Supplementary file 1 [file life-12-01804-s001.zip › Figure S3.pdf]

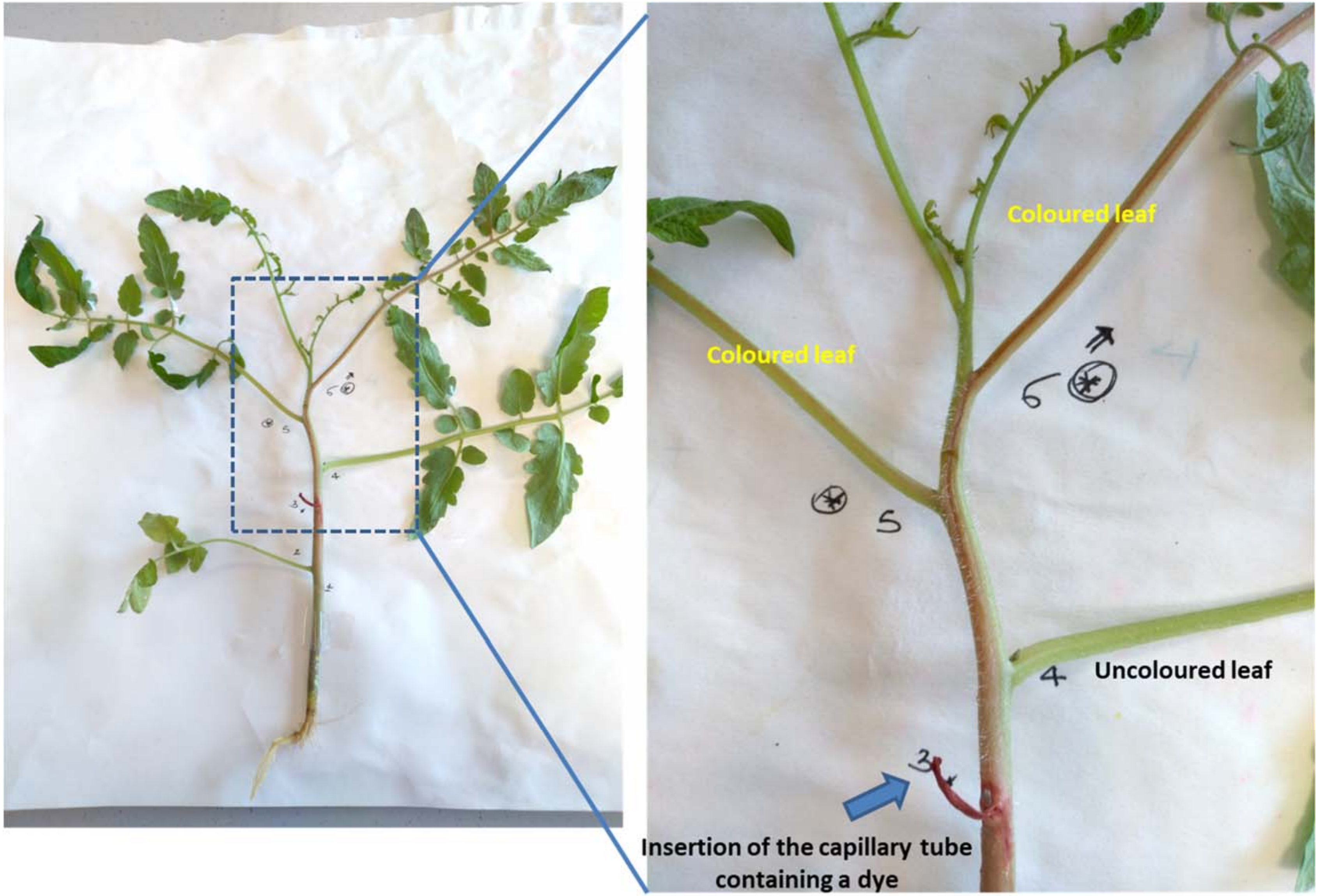

Figure S3 - Evaluation of the degree of vascular connectivity among leaves located in different position along the shoot. In order to evaluate the vascular constraints in systemic induction, we made a preliminary experiment to analyze how the different leaves of tomato plants are connected each one. The method consist of the insertion of a capillary tube containing a dye (Rhodamine-B) onto the petiole of the first true leaf. After 24 hr,

the amount of dye in each fully expanded leaf was visually scored as low, medium, or high. The results are that the leaf 1 is more connected to leaf 3 and much more to leaf 4. But non connection was observed with leaf 2.
