# Supplementary material for: Abiotic and Herbivory Combined Stress in Tomato: Additive, Synergic and Antagonistic Effects and Within-Plant Phenotypic Plasticity
Source: Life (Basel). 2022 Nov 7;12(11):1804. doi: 10.3390/life12111804 (PMC9699328; doi:10.3390/life12111804)
Supplement: Supplementary file 1 [file life-12-01804-s001.zip › Figure S4.pdf]

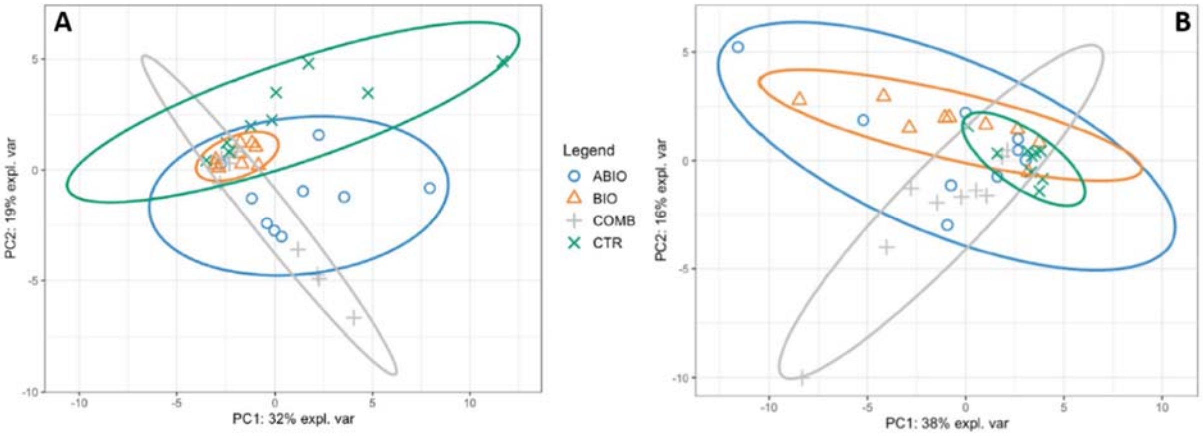

Figure S4 - Principal component analysis applied to volatiles emission data obtained from tomato leaves treated with different stress (ABIO, BIO, COMB) or not treated (CTR) for 3 (A) and 8 days (B) of exposure.
