# Supplementary material for: Abiotic and Herbivory Combined Stress in Tomato: Additive, Synergic and Antagonistic Effects and Within-Plant Phenotypic Plasticity
Source: Life (Basel). 2022 Nov 7;12(11):1804. doi: 10.3390/life12111804 (PMC9699328; doi:10.3390/life12111804)
Supplement: Supplementary file 1 [file life-12-01804-s001.zip › table S1.pdf]

Table S1 - Volatile organic compounds identified using gas chromatography/mass spectrometry (GC/MS) analysis by HS/SPME method in the leaves of the tomato plants treated with abiotic, biotic and combined stress. RT, retention time; KI, retention index.

common name	IUPAC name	Class	RT (min)	KI
(E) - 2 hexen - 1 ol	(E)-hex-2-en-1-ol	Alcohol	5,97	873
alpha pinene	2,6,6-trimethylbicyclo[3.1.1]hept-2-ene	monoterpene	7,36	939
beta pinene	6,6-dimethyl-2-methylidenebicyclo[3.1.1]heptane	monoterpene	8,22	981
Myrcene	7-methyl-3-methylideneocta-1,6-diene	monoterpene	8,61	993
alpha terpinene	1-methyl-4-propan-2-ylcyclohexa-1,3-diene	monoterpene	8,85	1018
(+)-4-Carene	4,7,7-Trimethylbicyclo[4.1.0]hept-2-ene	monoterpene	9,19	1022
o – cymene	1-methyl-2-propan-2-ylbenzene	monoterpene	9,51	1026
beta phellandrene	3-methylidene-6-propan-2-ylcyclohexene	monoterpene	9,59	1033
(E) beta ocimene	(3E)-3,7-dimethylocta-1,3,6-triene	monoterpene	9,98	1050
gamma terpinene	1-methyl-4-propan-2-ylcyclohexa-1,4-diene	monoterpene	10,3	1062
Terpinolene	1-methyl-4-propan-2-ylidenecyclohexene	monoterpene	11,05	1090
3-Hexen-1-ol, propanoate, (3Z)-	[(Z)-hex-3-enyl] propanoate	Ester	11,29	1103
Hexyl propionate	hexyl propanoate	Ester	11,34	1110
(2E)-2-Hexenyl propionate	[(E)-hex-2-enyl] propanoate	Ester	11,5	1112
1,3,7-Nonatriene, 4,8-dimethyl-, (3E)-	(3E)-4,8-dimethylnona-1,3,7-triene	hydrocarbon	11,67	1119
allo-ocimene	(4E,6E)-2,6-dimethylocta-2,4,6-triene	monoterpene	11,71	1133
Dodecane	dodecane	hydrocarbon	13,6	1200
methyl salicylate	methyl 2-hydroxybenzoate	Ester	13,69	1193
Dodecanal	dodecanal	Aldehydes	13,94	1204
Dodecane, 2,6,11-trimethyl-	2,6,11-trimethyldodecane	hydrocarbon	15,58	1275
Tridecane	tridecane	hydrocarbon	16,63	1302
delta elemene	(3R,4R)-4-ethenyl-4-methyl-1-propan-2-yl-3-prop-1-en-2-ylcyclohexene	sesquiterpene	17	1340
alfa copaene	(2R,6R)-1,3-dimethyl-8-propan-2-yltricyclo[4.4.0.02,7]dec-3-ene	sesquiterpene	17,89	1376
alfa cubebene	(5S,6R,7S,10R)-4,10-dimethyl-7-propan-2-yltricyclo[4.4.0.01,5]dec-3-ene	sesquiterpene	17,89	1344
beta elemene	(1S,2S,4R)-1-ethenyl-1-methyl-2,4-bis(prop-1-en-2-yl)cyclohexane	sesquiterpene	18,22	1393
alpha gurjunene	(1aR,4R,4aR,7bS)-1,1,4,7-tetramethyl-1a,2,3,4,4a,5,6,7b-octahydrocyclopropa[e]azulene	sesquiterpene	18,53	1410

Z beta caryophyllene	(1S,4E,9S)-4,11,11-trimethyl-8-methylidenebicyclo[7.2.0]undec-4-ene	sesquiterpene	18,6	1416
Longifolene	3,3,7-trimethyl-8-methylidenetricyclo[5.4.0.02,9]undecane	sesquiterpene	18,61	1404
E β-Caryophyllene	(1R,4E,9S)-4,11,11-trimethyl-8-methylidenebicyclo[7.2.0]undec-4-ene	sesquiterpene	18,88	1428
gamma elemene	(1S,2S)-1-ethenyl-1-methyl-4-propan-2-ylidene-2-prop-1-en-2-ylcyclohexane	sesquiterpene	19,06	1431
β-copaene	(1S,6S,7S,8S)-1-methyl-3-methylidene-8-propan-2-yltricyclo[4.4.0.02,7]decane	sesquiterpene	19,07	1431
alpha guauiene	(1S,4S,7R)-1,4-dimethyl-7-prop-1-en-2-yl-1,2,3,4,5,6,7,8-octahydroazulene	sesquiterpene	19,35	1439
aromadendrene	1,1,7-trimethyl-4-methylidene-2,3,4a,5,6,7,7a,7b-octahydro-1aH-cyclopropa[e]azulene	sesquiterpene	19,5	1445
Alloaromadendrene	1,1,7-trimethyl-4-methylidene-2,3,4a,5,6,7,7a,7b-octahydro-1aH-cyclopropa[e]azulene	sesquiterpene	19,52	1462
alpha humulene	(1E,4E,8E)-2,6,6,9-tetramethylcycloundeca-1,4,8-triene	sesquiterpene	19,61	1456
γ muurolene	(1S,4aS,8aR)-7-methyl-4-methylidene-1-propan-2-yl-2,3,4a,5,6,8a-hexahydro-1H-naphthalene	sesquiterpene	20,08	1477
γ gurjunene	1,4-dimethyl-7-prop-1-en-2-yl-1,2,3,3a,4,5,6,7-octahydroazulene	sesquiterpene	20,12	1477
germacrene D	(1E,6E,8S)-1-methyl-5-methylidene-8-propan-2-ylcyclodeca-1,6-diene	sesquiterpene	20,31	1481
beta cadinene	(1S,4aR,8aS)-4,7-dimethyl-1-propan-2-yl-1,2,4a,5,8,8a-hexahydronaphthalene	sesquiterpene	20,36	1500
alpha selinene	(3R,4aR,8aR)-5,8a-dimethyl-3-prop-1-en-2-yl-2,3,4,4a,7,8-hexahydro-1H-naphthalene	sesquiterpene	20,41	1500
delta cadinene	(1S,8aR)-4,7-dimethyl-1-propan-2-yl-1,2,3,5,6,8a-hexahydronaphthalene	sesquiterpene	21,01	1524
caryophyllene alcohol	4,4,8-trimethyltricyclo[6.3.1.02,5]dodecan-1-ol	Alcohol	21,87	1571
Dendrolasin	3-[(3E)-4,8-dimethylnona-3,7-dienyl]furan	Ether	22,08	1575
hexadecane	hexadecane	hydrocarbon	22,37	1600
Dioctyl ether	1-octoxyoctane	Ether	23,69	1660
