# Supplementary material for: Abiotic and Herbivory Combined Stress in Tomato: Additive, Synergic and Antagonistic Effects and Within-Plant Phenotypic Plasticity
Source: Life (Basel). 2022 Nov 7;12(11):1804. doi: 10.3390/life12111804 (PMC9699328; doi:10.3390/life12111804)
Supplement: Supplementary file 1 [file life-12-01804-s001.zip › Table S2.pdf]

Table S2 - Volatile organic compounds (Area %) from the leaves of tomato plants detected in at least two replicates out of three at each sampling time (t0, t1, t3 and t8) and treatments (ABIO, BIO, COMB and CTR).

N	Common name	t0				t1				t3				t8			
		ABIO	BIO	COMB	CTR	ABIO	BIO	COMB	CTR	ABIO	BIO	COMB	CTR	ABIO	BIO	COMB	CTR
1	(+)-4-Carene	1.161	2.199	1.508	2.438	1.916	2.054	1.300	2.062	1.570	2.081	0.872	1.882	1.321	1.898	1.443	1.717
2	(2E)-2-Hexenyl propionate	-	-	-	-	-	-	-	-	-	-	-	-	-	0.154	0.010	-
3	(E) - 2 hexen - 1 ol	4.689	10.781	2.015	8.795	2.270	4.651	7.857	8.242	1.035	4.897	5.640	11.923	0.558	13.908	8.854	12.541
4	(E) beta ocimene	0.066	0.510	0.280	0.682	0.584	0.252	1.945	5.035	0.603	0.401	0.925	0.401	0.966	0.348	0.780	0.168
5	1,3,7-Nonatriene, 4,8-dimethyl-, (3E)-	-	-	0.054	0.154	0.113	0.029	0.449	0.005	0.136	-	0.149	-	0.163	-	0.123	-
6	3-Hexen-1-ol, propanoate, (3Z)-	0.124	0.297	-	0.475	-	0.319	-	0.181	0.014	0.233	0.206	0.249	0.032	0.073	0.071	0.192
7	alfa copaene	0.087	0.018	0.031	0.036	0.073	0.040	0.032	0.040	0.059	0.021	0.080	0.015	0.026	0.048	0.033	0.049
8	alfa cubebene	-	0.005	0.002	-	0.002	-	0.002	-	-	-	-	-	0.002	-	-	-
9	Alloaromadendrene	-	0.002	0.006	0.003	-	-	-	-	0.005	-	-	-	-	-	-	-
10	allo-ocimene	0.094	0.146	0.135	0.160	0.207	0.135	0.433	0.129	0.190	0.065	0.221	0.189	0.222	0.056	0.167	0.117
11	alpha guauiene	0.135	0.138	0.248	0.073	0.182	0.037	0.281	0.073	0.134	0.049	0.221	0.029	0.084	0.024	0.108	0.023
12	alpha gurjunene	-	-	0.008	-	-	-	-	-	-	-	-	-	-	-	-	-
13	alpha humulene	0.948	1.486	2.592	0.724	2.089	0.569	2.882	0.821	1.896	0.713	1.815	0.340	0.707	0.427	1.204	0.363
14	alpha pinene	1.962	2.2232	1.720	2.613	2.596	2.630	1.988	5.089	2.333	2.488	1.968	4.479	2.521	2.233	2.249	2.519
15	alpha selinene	-	-	-	-	-	-	-	-	-	-	-	-	-	-	0.003	-
16	alpha terpinene	23.117	22.598	21.464	27.941	18.196	26.618	17.120	22.316	22.055	20.163	19.942	22.084	24.387	23.187	21.425	24.561
17	aromadendrene	-	-	0.100	0.033	0.114	-	0.168	0.017	0.149	0.147	0.094	0.013	0.025	0.004	0.084	0.045
18	beta cadinene	0.086	0.084	0.137	0.063	0.083	0.034	0.279	0.043	0.109	0.017	0.197	0.008	0.088	0.018	0.091	0.047
19	beta elemene	0.257	0.130	0.257	0.126	0.213	0.018	0.337	0.056	0.168	0.053	1.137	0.019	0.093	0.025	0.137	0.112
20	beta phellandrene	51.056	39.824	42.917	44.314	19.965	52.715	38.307	45.478	48.506	57.142	41.944	46.486	56.737	48.859	46.358	49.086
21	beta pinene	5.432	2.481	2.781	1.851	1.043	2.502	1.922	2.654	2.087	2.154	1.889	6.098	2.602	2.638	1.890	2.323

22	caryophyllene alcohol	-	-	0.007	-	-	0.032	1.601	-	-	0.007	-	-	-	0.004	0.002	-
23	delta cadinene	-	-	0.035	-	0.006	-	0.007	-	-	-	-	-	0.007	-	0.017	-
24	delta elemene	0.836	1.003	2.875	0.584	2.285	0.308	3.013	0.537	1.981	0.582	2.929	0.287	1.035	0.225	1.861	0.439
25	Dioctyl ether	-	-	-	-	-	-	-	-	-	-	-	0.099	-	-	-	0.449
26	dodecanal	0.111	-	-	-	-	-		-	-	-	-	-	-	-	-	-
27	Dodecane	0.118	-	-	-	-	-	-	-	-	-	-	-	-	-	-	-
28	Dodecane, 2,6,11-trimethyl-	0.158	0.050	0.022	0.059	0.023	0.062	0.012	0.044	0.020	0.072	0.045	0.016	0.018	0.004	0.017	0.050
29	E β-Caryophyllene	5.271	10.632	15.863	4.919	12.195	4.189	11.917	4.980	11.114	5.483	10.191	4.347	4.550	2.705	7.376	2.304
30	hexadecane	0.088	-	-	0.015	-	-	-	-	-	-	-	-	-	-	-	-
31	Dendrolasin	-	0.202	0.189	0.215	0.133	0.520	0.130	-	0.553	0.124	0.843	0.084	0.162	0.260	0.156	0.846
32	gamma elemene	0.261	0.184	0.580	0.282	0.662	0.108	0.581	0.206	0.485	0.163	0.141	0.049	0.075	0.078	0.240	0.056
33	gamma terpinene	0.117	0.343	0.207	0.509	0.423	0.467	0.181	0.345	0.344	0.487	0.437	0.470	0.355	0.299	0.175	0.392
34	germacrene D	-	-	0.027	-	-	-	-	-	0.007	-	-	-	0.004	-	0.003	-
35	hexyl propionate	-	0.098	-	-	-	-	-	-	-	-	-	-	0.040	0.092	0.010	-
36	Longifolene	-	0.003	-	-	-	-	-	-	-	-	-	-	-	-	-	-
37	methyl salicylate	-	-	-	-	-	-	-	-	-	-	4.159	-	0.332	0.534	0.913	-
38	myrcene	3.089	4.035	3.299	2.183	3.791	1.344	6.460	1.280	3.735	2.015	3.459	1.620	2.424	1.588	3.700	1.159
39	o - cymene	-	-	-	-	0.207	-	-	-	0.215	-	-	-	-	-	-	0.073
40	terpinolene	0.306	0.389	0.348	0.396	0.416	0.277	0.474	0.246	0.272	0.368	0.290	0.348	0.361	0.267	0.329	0.282
41	tridecane	0.072	-	-	0.024	-	-	-	-	-	-	-	-	-	-	-	-
42	Z beta caryophyllene	0.021	-	0.088	0.055	0.098	0.048	0.092	0.035	0.056	0.044	0.084	0.008	0.015	0.010	0.050	0.052
43	β-copaene	-	-	-	-	-	-	-	0.008	-	-	-	-	-	-	-	-
44	γ gurjunene	-	-	0.004	0.001	0.007	-	0.019	-	0.014	-	-	0.001	0.010	-	-	0.001
45	γ muurolene	0.081	0.056	0.197	0.029	0.098	0.001	0.207	0.056	0.137	0.005	0.113	-	0.068	0.015	0.115	0.004
