# Supplementary material for: Abiotic and Herbivory Combined Stress in Tomato: Additive, Synergic and Antagonistic Effects and Within-Plant Phenotypic Plasticity
Source: Life (Basel). 2022 Nov 7;12(11):1804. doi: 10.3390/life12111804 (PMC9699328; doi:10.3390/life12111804)
Supplement: Supplementary file 1 [file life-12-01804-s001.zip › Table S3.pdf]

Table S3 - Permanova results of the VOC emission of tomato plants exposed to different stress (Treatments) and time of exposure (Time).

	Df	Sum of square	R2	F	Pr(>F)
**Treatments**	3	0.8661	0.06288	3.3117	0.003**
**Time**	1	0.2228	0.01618	2.5562	0.048*
**Treatments x Time**	3	0.8291	0.06020	3.1704	0.007**
**Residual**	136	11.8555	0.86075		
**Total**	143	13.7736	1.00000		
