# Supplementary material for: Abiotic and Herbivory Combined Stress in Tomato: Additive, Synergic and Antagonistic Effects and Within-Plant Phenotypic Plasticity
Source: Life (Basel). 2022 Nov 7;12(11):1804. doi: 10.3390/life12111804 (PMC9699328; doi:10.3390/life12111804)
Supplement: Supplementary file 1 [file life-12-01804-s001.zip › Table S4.pdf]

Table S4 - PERMANOVA pairwise comparison between treatments (ABIO, BIO, COMB and CTR) within each time of exposure (0, 1, 3 and 8 days).

**Pairs**	**F. Model**	**R2**	**P value**	**P adjusted**
ABIO 0 vs BIO 0	2.36754570	0.128898316	0.065	0.15230769
ABIO 0 vs COMB 0	1.73298156	0.097726462	0.159	0.23850000
ABIO 0 vs CTR 0	1.91671243	0.106979025	0.145	0.22894737
ABIO 1 vs BIO 1	2.25116265	0.123343520	0.099	0.19161290
ABIO 1 vs COMB 1	1.68353749	0.095203660	0.150	0.23376623
ABIO 1 vs CTR 1	1.69383507	0.095730240	0.143	0.22880000
ABIO 3 vs BIO 3	1.06694916	0.062515517	0.289	0.35030303
ABIO 3 vs COMB 3	1.57385814	0.089556780	0.197	0.26696629
ABIO 3 vs CTR 3	2.05710766	0.113922323	0.136	0.22054054
ABIO 8 vs BIO 8	1.65593317	0.093789049	0.190	0.26696629
ABIO 8 vs COMB 8	2.22174702	0.121928321	0.090	0.18620690
ABIO 8 vs CTR 8	4.25785175	0.210182787	0.018	0.06967742
BIO 0 vs COMB 0	0.72193155	0.043172737	0.473	0.51459459
BIO 0 vs CTR 0	0.24011804	0.014785486	0.831	0.83798319
BIO 1 vs COMB 1	2.42871064	0.131789504	0.066	0.15230769
BIO 1 vs CTR 1	1.31246112	0.075810199	0.259	0.32041237
BIO 3 vs COMB 3	1.65423201	0.093701726	0.186	0.26696629
BIO 3 vs CTR 3	1.95860104	0.109062005	0.122	0.20054795
BIO 8 vs COMB 8	4.73388820	0.228316472	0.024	0.08228571
BIO 8 vs CTR 8	9.79313456	0.379679893	0.004	0.04000000
COMB 0 vs CTR 0	0.70477038	0.042189767	0.473	0.51459459
COMB 1 vs CTR 1	3.05268470	0.160223336	0.027	0.08756757
COMB 3 vs CTR 3	5.21797207	0.245922280	0.006	0.04235294
COMB 8 vs CTR 8	9.03428123	0.360876398	0.001	0.04000000
