# Supplementary figures and images for: Abiotic and Herbivory Combined Stress in Tomato: Additive, Synergic and Antagonistic Effects and Within-Plant Phenotypic Plasticity
Source: Life (Basel). 2022 Nov 7;12(11):1804. doi: 10.3390/life12111804 (PMC9699328; doi:10.3390/life12111804)

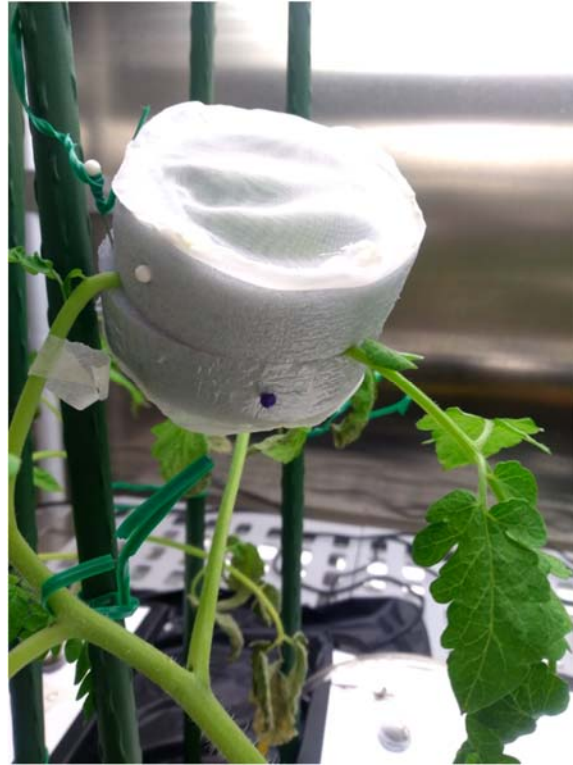

**Figure S2** - Trap for *Tuta absoluta*'s larva.

Supplement: Supplementary file 1 [file life-12-01804-s001.zip › Figure S2.pdf]
